# Carbon availability affects already large species-specific differences in chemical composition of ectomycorrhizal fungal mycelia in pure culture

**DOI:** 10.1007/s00572-023-01128-2

**Published:** 2023-10-12

**Authors:** Petra Fransson, A. H. Jean Robertson, Colin D. Campbell

**Affiliations:** 1 Department of Forest Mycology and Plant Pathology, Uppsala BioCenter, Swedish University of Agricultural Sciences, PO Box 7026, SE-750 07 Uppsala, Sweden; 2 The James Hutton Institute, Craigiebuckler, Aberdeen, AB15 8QH Scotland

**Keywords:** Carbohydrates, Cell wall composition, Fourier transform infrared spectroscopy, Mycelia, Soil organic carbon, C:N ratio

## Abstract

Although ectomycorrhizal (ECM) contribution to soil organic matter processes receives increased attention, little is known about fundamental differences in chemical composition among species, and how that may be affected by carbon (C) availability. Here, we study how 16 species (incl. 19 isolates) grown in pure culture at three different C:N ratios (10:1, 20:1, and 40:1) vary in chemical structure, using Fourier transform infrared (FTIR) spectroscopy. We hypothesized that C availability impacts directly on chemical composition, expecting increased C availability to lead to more carbohydrates and less proteins in the mycelia. There were strong and significant effects of ECM species (*R*^2^ = 0.873 and *P* = 0.001) and large species-specific differences in chemical composition. Chemical composition also changed significantly with C availability, and increased C led to more polysaccharides and less proteins for many species, but not all. Understanding how chemical composition change with altered C availability is a first step towards understanding their role in organic matter accumulation and decomposition.

**Supplementary Information:**

The online version contains supplementary material available at 10.1007/s00572-023-01128-2.

## Introduction

The majority of plants form symbiosis with root-associated fungi, and these fungi play critical roles in carbon (C) and nutrient cycling. In northern forest biomes, ectomycorrhizal (ECM) fungi belonging to Ascomycota and Basidiomycota are most abundant (Brundrett 2009), and the influence of ECM fungi on nutrient uptake is well documented. The mycorrhizal contribution to soil organic matter processes is receiving increased attention, as we are beginning to recognize the importance of the ECM mycelium for soil C accumulation and as potential drivers of organic matter decomposition and related losses of C (Adamczyk et al. 2019; Averill and Hawkes 2016; Godbold et al. 2006; Lindahl et al. 2021; Lindahl and Tunlid 2015; Zak et al. 2019). Belowground litter inputs may be more important than aboveground litter input in many ecosystems (Kätterer et al. 2011); Clemmensen and colleagues (2013) suggested that organic layers grow from below through the continuous addition of recently fixed C to the organic matter profile in the form of remains from roots and associated mycelium. Plants allocate up to 20% of their photosynthetically fixed C belowground to mycorrhiza (Hobbie 2006), resulting in a large standing biomass of extraradical mycelia in soils. Up to 1.2 kg mycelia may be produced per hectare every day in young Scots pine forest (Hagenbo et al. 2017), and the ECM mycelial production in coniferous forests has been estimated to be several hundred kilogram per hectare per year in the upper part of the soil (Ekblad et al. 2013), which is in the same range as the biomass of fine roots. Mycelial turnover will depend on chemistry, C:N stoichiometry, and morphology as well as decomposer community activity and physical protection by soil (Fernandez et al. 2016); therefore, organic matter accumulation will be affected by which fungal species are present in the community, and what their chemical compositions are. For example, the presence of recalcitrant chemical constituents such as melanin, oxidized polymers of phenolic materials that require oxidative enzymes for degradation, will potentially decrease decomposition rates and at the same time increase organic matter accumulation (Fernandez et al. 2016), while mycelia with high nitrogen (N) concentration are subject to higher decomposition rates (Koide and Malcolm 2009). The chemical composition of ECM mycelia is thus key to a better understanding of which factors may drive soil organic matter accumulation.

The fungal cell wall is central for the chemical composition of mycelia since up to 50% of the fungal biomass is found in the cell wall (Ruiz-Herrera 1991). This represents a considerable metabolic investment, and cell wall composition of different fungi is diverse and varies depending on species, genotype, age and environment (Bowman and Free 2006; Feofilova 2010; Wessels 1994). Although the composition of non-cell wall components also can alter due to environmental conditions (e.g., heat; Tereshina et al. 2011), the cytoplasmic fraction of fungal tissue does not likely play a significant role in the decomposition of ECM mycelia since cytoplasm is moved to active parts of the mycelium at senescence resulting in vacuolated hyphae (Cooke and Rayner 1984). Furthermore, cytoplasm is highly labile and readily taken up by decomposers (Nakas and Klein 1979). The composition of the cell wall was suggested to exert the most control on long-term decomposition of ECM fungal necromass (Fernandez et al. 2016), and melanin and N concentrations appear to be key biochemical controllers of decomposition in fungal litter (Fernandez and Koide 2014). The major cell wall component is polysaccharides (up to 80–90% of dry mass; Bartnicki-Garcia 1968), but there are also lipids (up to 3%; Feofilova 2010) and glycoproteins (15–30% of dry mass) with varying content, function, and chemistry (Fernandez et al. 2016). The main structural elements are chitin microfibrils (the fungal-specific crystalline homopolymer of linked N-acetyl-D-glucosamine residues) and β-glucans (homopolymers of glucose making up the central core of the cell wall), embedded in an amorphous gel-like matrix (Feofilova 2010). Chitin, an important source of N (Leake and Read 1990), is the structural equivalent to plant lignin and concentrations range from ca. 1 to 10% in ECM fungi (Ekblad et al. 1998; Fernandez and Koide 2012), while β-glucans constitute approximately 50–60% of dry mass (Kapteyn et al. 1999). Cell walls also consist of monosaccharides (glucose, mannose, and xylose), amino acids, and hydrophobins, which are secretory surface proteins important for ECM formation (Fernandez et al. 2016), hydrophobicity, and adherence to substrata (Feofilova 2010). Older parts of the mycelia have increased levels of lipids and pigments such as melanins, contributing to surface properties and protection against stressors. Since we do not know how ECM chemical composition varies between species or in relation to different growth conditions, a better understanding of their mycelial properties would improve our understanding of how different ECM communities may potentially feedback on soil organic matter processes.

Fourier transform infrared (FTIR) spectroscopy is a widely used technique which gives the overall chemical composition of a sample, including both organic and inorganic components. The FTIR spectrum of a sample arises from an interaction between IR radiation and the vibrations of molecules present within the sample. IR radiation is absorbed at frequencies which directly relate to the frequencies of vibrations of functional groups present in the sample, and the IR absorption bands observed relate to all the constituents which make up its chemical composition. The technique is thus capable of distinguishing the principal chemical classes in, e.g., cell walls, and can therefore provide a unique insight into differences in chemical composition between samples of different species or for samples of a single species grown under different conditions. The technique has been extensively used for bacterial cell walls, and to a growing extent to examine fungi. Studies are published on changes in chemical composition during hyphal growth and morphogenesis (Adt et al. 2006; Jilkine et al. 2007; Szeghalmi et al. 2007), detection of fungi in different plant substrates (Kos et al. 2002; Naumann et al. 2005), differentiation of plant pathogen isolates, molds, and fungi in food industry (Lecellier et al. 2015; Pomerantza et al. 2014; Salman et al. 2012; Shapaval et al. 2010), and processes involving fungi such as substance transformations, decomposition, and carbon sequestration (Jeewani et al. 2021; Mrnka et al. 2020). The method has also been suggested to distinguish between fungal species (Adt et al. 2006; Essendoubi et al. 2005; Fischer et al. 2006; Lecellier et al. 2015; Naumann et al. 2005; Rellini et al. 2009; Santos et al. 2010). Most studies of species’ differences have used fungal isolates grown under controlled growth conditions (e.g., Lecellier et al. 2014; Naumann et al. 2005) to avoid alterations of spectra due to fungal responses to environmental changes (Pena et al. 2014). However, for mycorrhizal fungi, this technique had not been commonly used. In a study by Pena et al. (2014), the suitability and limitations of FTIR for the distinction of ECM roots in field samples were tested. The use of in situ collected material showed that environmental variability did not limit discrimination between species. It is worth noting though that ECM roots of the four species tested are morphologically very distinct from each other. The knowledge about the chemical composition in ECM fungal mycelia based on FTIR analysis is thus still very limited.

Since ECM fungi are supported by belowground C allocation of plant photoassimilates (Hobbie 2006), and C availability is one key factor determining mycelial production (Ekblad et al. 2013), mycelial production will be affected by changes in C availability. We hypothesized that C availability impacts not only the production of mycelia but also directly on the chemical composition of mycelia and expect an increased C availability to lead to more carbohydrates and less proteins. This was tested by growing 16 different ECM fungal species in liquid media at three different C:N ratios, after which the chemical composition was determined using FTIR analysis. By using a pure culture setup, we could control for variation due to growth environment. Our secondary aim was to determine if FTIR spectroscopy could be used to distinguish between different ECM species and what this tells us about genetically determined differences. We addressed the following questions: (1) Are ECM fungal species different in chemical composition under the same growth conditions? (2) Can ECM fungal species be distinguished from one another under the same growth conditions by their chemical composition? (3) What are the potential differences in chemical composition among ECM species related to structurally? (4) How does increased C availability affect chemical composition? (5) Are potential differences in chemical composition among ECM species related to putative ecological role and/or functional traits? (6) How useful is FTIR as a method to distinguish ECM species and to explain differences in chemical composition? Understanding differences in chemical composition of ECM fungi, and how they change with altered C availability, is a key step towards understanding their role in organic matter accumulation and decomposition.

## Materials and methods

### Fungal strains and experimental conditions

A total of 19 fungal isolates, covering 16 different ECM species, were used in the study (Table 1). Identity of these strains was confirmed by ITS sequence analysis using standard techniques (see Fransson and Johansson 2010). DNA was extracted according to Gardes et al. (1991), and polymerase chain reactions (PCR) were carried out using the primers ITS1 and ITS4 as described by White et al. (1990). Fungal cultures were kept in darkness at 25 °C on 25 mL agar plates containing cellophane covered Modified Melin Norkrans (MMN) medium at pH 5.5, with the following modifications: 2.5 g L^−1^ glucose, 10 g L^−1^ malt extract, 15 g L^−1^ agar (Marx 1969). Fungal inoculum (three pieces à 5 × 5 mm) were cut from the actively growing mycelial front of each plate with a scalpel and placed in 90-mm Petri dishes containing 30 mL liquid Basal Norkrans (BN) medium (Norkrans 1950) at pH 4.5. Care was taken to move the fungal inoculum without any agar and cellophane; however, the isolate of *Laccaria laccata* dissolved the cellophane which made it difficult to avoid the agar completely for this particular species. The spectra for replicates of this species did match closely; suggesting contamination was not an issue. Petri dishes were sealed using Parafilm®.

**Table 1:** Ectomycorrhizal fungal isolates used to study chemical composition after growth in liquid medium under three different C:N ratios (10:1, 20:1, and 40:1), and classification of fungal species according to their putative ecological role and traits

**ECM fungal isolate**	**Isolate code**	**GenBank accession No**	**Exploration type** ^f^	**Order**	**Hydrophobicity** ^h^	**N tolerance** ^l^	**Succession** ^p^
*Amanita muscaria* (L.:Fr.) Hook	UP3	DQ179118	Medium-distance/smooth	Agaricales	Hydrophobic	High	Early
*Amphinema byssoides* (Pers.:Fr.) J. Erikss	A705	EF493272	Medium-distance/fringe	Atheliales	Hydrophobic	Low(-high) ^m^	Early
*Cenococcum geophilum* Fr. ^a^	Ve-95–12	DQ179119	Short-distance	Dothideomycetes ^g^	Hydrophilic	Low(-high) ^m^	Multi
*Cortinarius glaucopus* (Sch.:Fr) Fr	UP21	DQ179120	Medium-distance/fringe	Agaricales	Hydrophobic	Low	Late
*C. scaurus* (Fr.: Fr.) Fr	UP22	-	Medium-distance/fringe	Agaricales	Hydrophobic	Low	Late
*Hebeloma velutipes* Bruchet	UP184	AF432845 ^d^	Short-distance	Agaricales	Hydrophobic ^i^	High ^n^	Early-mid
*Hebeloma sp.*	Sp 1	-	Medium-distance/fringe	Agaricales	Hydrophilic	High	Early-mid
*Laccaria bicolor* (Maire) Orton	CRBF581	DQ179121	Medium-distance/fringe	Agaricales	Hydrophilic	High	Early
*L. laccata* (Scop.:Fr.) Berk. and Br	Sk33	-	Medium-distance/fringe	Agaricales	Hydrophilic	High	Early-mid
*Paxillus involutus* (Batsch:Fr.) Fr	G05	DQ179126	Long-distance	Boletales	Hydrophobic	High	Late?
*“Piceirhiza bicolorata”* ^a, b^	ARON2938.S	AJ430152 ^e^	Contact	Helotiales	Hydrophilic/hydrophobi ^j^	Low ^o^	Late
*Piloderma byssinum* (P. Karst.) Jülich ^a^	UP185	DQ179124	Medium-distance/fringe	Atheliales	Hydrophobic	Low	Late
*P. fallax* (Liberta) Stalpers ^a^	UP113	DQ179125	Medium-distance/fringe	Atheliales	Hydrophobic	Low	Multi
*Rhizopogon roseolus* (Corda) Th. M. Fr	UP175	DQ179127	Long-distance	Boletales	Hydrophobic ^k^	Low	Late
*Suillus bovinus* (L.:Fr.) Roussel	BL	DQ179128	Long-distance	Boletales	Hydrophobic	Low	Late
	UP63^c^	-					
	UP592	EF493250					
*S. variegatus* (Sw.:Fr.) O. Kuntze	UP60	DQ179130	Long-distance	Boletales	Hydrophobic	Low	
	UP597	EF493256					

^a^Isolated from ectomycorrhizal root tip

^b^Mycorrhizal root tip name (Brand et al. 1992) corresponding to the characteristic black mycorrhizas formed by ascomycetous fungi in the *Rhizoscyphus ericae* species complex, now referred to as *Meliniomyces bicolor* Hambleton and Siegler, sp. nov. (Hambleton and Sigler 2005)

^c^Isolated from fruitbody by P. Fransson

^d^Sequenced by Anna Rosling (Rosling et al. 2004)

^e^Sequenced by Trude Vrålstad (Vrålstad et al. 2000)

^f^Pattern of mycelial differentiation according toAgerer (2001)

^g^Taxonomic level set to class

^h^Accordning toHobbie and Agerer (2010)

^i^The genus *Hebeloma* classified as uncertain hydrophobicity according to Hobbie and Agerer (2010); the isolate displays hydrophobic properties in liquid culture

^j^Predominantly hydrophilic according to Agerer (2006); the isolate displays both hydrophilic and hydrophobic properties in liquid culture

^k^The genus *Rhizopogon* has either hydrophobic or hydrophilic rhizomorphs according to Hobbie and Agerer (2010); the isolate displays hydrophobic properties in liquid culture

^l^Based on where species from ECM root tips were found predominantly over a N deposition gradient according toLilleskov et al. (2002)

^m^Low N level dominating species according to Lilleskov et al. (2002), but frequently occurring in nurseries (Menkis et al. 2005; Rudawska et al. 2006) and after fertilization (Fransson et al. 2000)

^n^Intermediate N level dominating species according to Lilleskov et al. (2002), but the genera is frequently occurring in nurseries (Rudawska et al. 2006)

^o^*Meliniomyces* sp. decrease in abundance with increasing N availability (Kjøller et al. 2012; Dean et al. 2014)

^p^Successional stage, adapted from Deacon and Fleming (1992) and Twieg et al. (2007), and references therein

To test for potential effects of C availability all fungi were grown in duplicates at 25 °C for 3 weeks at three different C:N ratios: 10:1, 20:1, and 40:1, containing a constant N content of 0.236 g L^−1^ (NH_4_)_2_SO_4_ and a C content of 1.25, 2.5, and 5 g L^−1^ glucose, respectively. The C:N ratios were chosen based on the direct measurements of field collected mycelia reporting values of ca. 20:1 (Wallander et al. 2003), and in effect moving from conditions of C limitation (C:N 10:1) to conditions of N limitation (C:N 40:1). At harvest, fungal biomass was collected on muslin fabric, washed with 50 mL double distilled H_2_O and freeze-dried using a ScanVac™ CoolSafe 55–9 freeze drier (ScanLaf A/S, Denmark). Biomass dry weight was recorded, and samples were ground to a fine powder using a ball mill.

### FTIR spectroscopy

Spectral characterization of fungal samples was performed by FTIR spectroscopy. IR spectra were recorded on a Bruker Vertex 70 FTIR spectrometer (Bruker, Ettlingen, Germany) fitted with a potassium bromide beam splitter and a deutroglycine sulfate detector for two replicates of each species. A Diamond Attenuated Total Reflectance (DATR) sampling accessory, with a single reflectance system, was used to produce “transmission-like” spectra, then the IR transmittance spectra were converted to absorbance spectra using the spectral software which applies the formula A = log_10_T where A is absorbance and T is transmittance. Samples were placed directly on a DATR/KRS-5 crystal, and a flat tip powder press was used to achieve even distribution and contact. Spectra were acquired by averaging 200 scans at 4 cm^−1^ resolution over the range 4000–370 cm^−1^. A correction was made to the ATR spectra to allow for differences in depth of beam penetration at different wavelengths, using OPUS software (Bruker, Ettlingen, Germany, version 6.0). The spectra were also baseline corrected. No correction was required for water vapor and CO_2_ as the spectrometer is continuously flushed with dry air. Technical replication formed part of the performance checks for the FTIR instrument and method used. FTIR data was normalized by subtraction of the minimum value and subsequent division by the average of all data points per sample prior to statistical analyses. FTIR raw data has been deposited in Dryad (Fransson et al. 2023).

### Statistics

FTIR data were analyzed by principal components analysis (PCA) using Genstat for Windows (8th edition, VSN International, UK), followed by canonical variate analysis (CVA) of the PC scores using CAP (Community Analysis Package: a multipurpose package for community data, including classification and ordination) software (see Anderson 2004; Anderson and Willis 2003) for visualization of data. Data was standardized using David Hirst’s standardization (subtract mean and divide by standard deviation on a per sample basis). The PCA step was used to reduce the number of variables for CVA so that the sample number exceeded the number of variates for CVA, and the minimum numbers of PC scores needed to explain more than 95% of the total data variability were used for CVA. The adjusted loadings (or coefficients of linear discriminants for each absorbance value) for the first two original variates were plotted to indicate informative regions of the FTIR spectra. To test for overall treatment effects and partition the variation in FTIR spectra depending on ECM species and C:N ratio, permutational multivariate analysis of variance (perMANOVA) was run based on the first two variates, with Euclidean distance and 999 permutations using the adonis2 function from R (R core team 2023) Vegan package (Oksanen et al 2022). Since the treatments *Piloderma fallax* and *Suillus variegatus* UP60 were not fully replicated, they were excluded from the perMANOVA. Term position was changed to ensure that order does not matter, and in a second analysis, biomass was added as a covariate term (additive). Additionally, fungal species were classified according to their putative ecological role and/or according to functional traits (Table 1), including exploration type (pattern of mycelial differentiation; Agerer 2001), hydrophobicity (Hobbie and Agerer 2010), N tolerance (based on where ECM root tips were found along a N deposition gradient; Lilleskov et al. 2002), and successional stage (based on when during forest succession the species is preferentially found, adapted from Deacon and Fleming 1992). These classifications into groups (exploration types: contact, short-distance, medium-distance smooth, medium-distance/fringe and long-distance; hydrophobicity: hydrophobic, hydrophilic and hydrophobic/hydrophilic; N tolerance: low, low(-high), and high; and successional stage: early, early-mid, late, and multi) were used to potentially explain the variation in chemical composition. The Multi Response Permutation Procedure (MRPP) was applied to test whether there was a significant difference between the groups, using Euclidean distance and 999 permutations in the R-package Vegan (Oksanen et al 2022). MRPP provides a *P* value and an *A* value that is a measure of effect size, representing homogeneity within the group compared to that expected randomly. For example, perfect homogeneity in the group gives *A* = 1 whereas *A* values between 0 and 1 indicate that heterogeneity between the groups is more than that expected by chance. As a proxy for mycelial C:N ratio, we semi-quantitatively compared the relative height of the main C-O stretching vibration (~ 1030 cm^−1^, associated with polysaccharides) to the relative height of the amide bands (amide I ~ 1650, amide II ~ 1550 cm^−1^, associated with N) for *Amanita muscaria* and *Laccaria bicolor*. Two-way ANOVA was used to test for effect of fungal species and C availability (C:N ratios 10:1, 20:1, and 40:1) on biomass dry weights, using Minitab 16.0 (Minitab Inc., State College, PA, USA). When interpreting the IR spectrum of a sample as a “chemical fingerprint,” the important thing to note, when comparing spectra (e.g., biological replicates), is whether the same bands are present (no additional or missing bands) and if the bands are present with comparable relative intensities. This would indicate a very similar chemical composition. Furthermore, the reproducibility of ECM fungal spectra was evaluated based on the average coefficient of variation for the two replicate IR spectra for each treatment, after first calculating the coefficients of variation (standard deviation/sample average) for all individual wavenumbers (*n* = 1894). The lower the coefficient of variation the higher is the reproducibility. Three treatments without replicates were excluded (*P. fallax* C:N 20:1, and *S. variegatus* 20:1 and 40:1). Outliers were excluded from the calculation of averages when the coefficients of variation for individual wavenumbers exceeded 240. This was applicable to seven treatments in total, where either one (six treatments) or two individual wavenumbers (one treatment) were affected (see Table S1 for details).

## Results

### Characteristics and reproducibility of the fungal FTIR spectrum

**Fig. 1:**
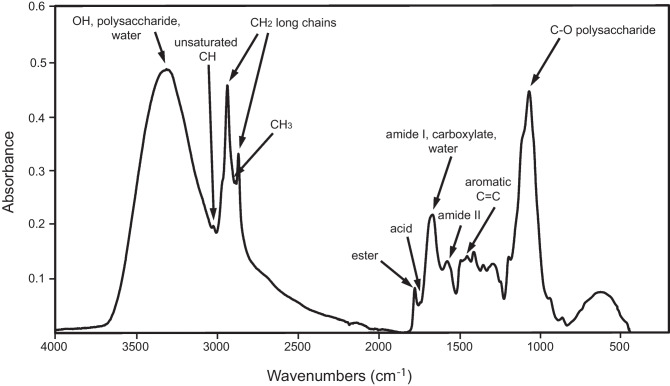
Characteristic bands of the major biochemical descriptors in ECM fungal FTIR spectra; based on fungal mycelia (*P. bicolorata*) grown in liquid culture at C:N ratio 20:1, with an inorganic N source ((NH_4_)_2_SO_4_)

In the case of ECM samples, the main constituents of the cell walls typically include polysaccharides, lipids, proteins, chitin, monosaccharides, amino acids, and β-glucans. The FTIR spectrum of the fungal mycelium (Fig. 1) was characterized by a strong, broad OH stretching absorbance band at ~ 3300 cm^−1^ and a strong C-O stretching absorbance band at 1030 cm^−1^ which both relate to the polysaccharides, present in relatively high proportions. Sharp CH_2_ stretching absorbance bands at 2920 and 2850 cm^−1^ are indicative of long chain C compounds, or waxy compounds, such as alkanes, acids, or esters. The ester functional group can be detected by an absorption band at around 1740 cm^−1^, with acids at a lower frequency, closer to 1710 cm^−1^. Amide functional groups (amide I ~ 1650 cm^−1^ and amide II ~ 1545 cm^−1^), which can arise from amino acids, chitin, or proteins, also form a distinctive part of the fungal spectrum. An NH stretching peak at 3260 cm^−1^ may be detectable but is sometimes concealed by the broad OH stretch. Water contributes to the broad OH stretching absorbance band and a related OH bending band at ~ 1630 cm^−1^. This band often overlaps with the amide I band, as does a band due to a carboxylate functional group. Other features, such as the presence of aromatic stretching absorbance bands (~ 1510 cm^−1^), can indicate the presence of phenolic compounds, e.g., melanin. In addition, CH stretches for unsaturated CH or for the CH_3_ functional group can sometimes be detected (3010 and 2870 cm^−1^, respectively) in the fungal spectrum. Reproducibility of fungal spectra was high, and the average coefficient of variation when comparing replicates within treatments (ECM species × C:N ratio) ranged between 0.05 and 15.4% (Table S1), with 48 out of 54 treatments below 10%. The replicate spectra for each of the specific C:N ratios for *Rhizopogon roseolus* could be almost exactly overlaid (Fig. S1), indicating how close their chemical compositions were. This was also the case for each of the other species studied (not shown).

### Ectomycorrhizal fungal species differences in chemical composition under the same growth conditions

**Fig. 2:**
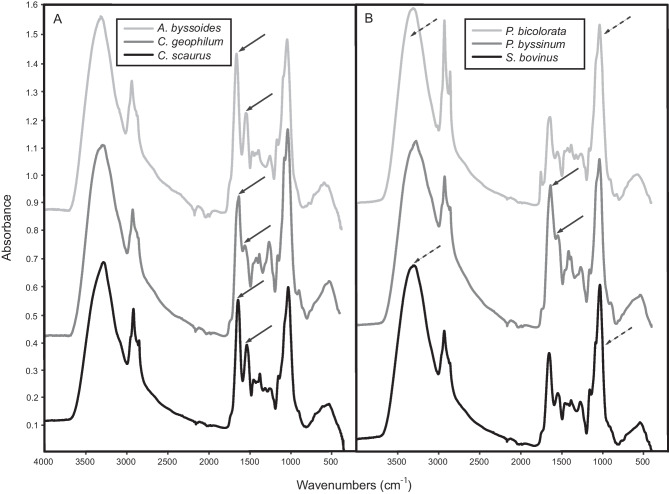
Species variation in FTIR spectra for different ECM fungal species, showing (A) *A. byssoides*, *C. geophilum* and *C. scarus*, and (B) *P. bicolorata*, *P. byssinum* and *S. bovinus*. Mycelia were grown in pure culture at C:N ratio 20:1 with an inorganic N source ((NH_4_)_2_SO_4_). Absorbance (spectral signals) was normalized to give relative absorbance/abundance, and spectra are shown off set. Black arrows in **a** indicate high amide/protein content in relation to the polysaccharide. Black arrows in **b** indicate the intense amide peaks seen in *P. byssinum*, and dashed arrows indicate a greater proportion of polysaccharide relative to amide/protein

The IR spectra of different ECM fungal species, grown under the same conditions, showed noticeable differences from each other, as illustrated in Fig. 2 (see Fig. S3 for spectra plotted overlaid). All consisted of similar components, e.g., predominantly polysaccharide and amide/protein, but with different relative proportions and some other differences such as the extent to which waxy compounds were present, or how distinct bands relating to ring linkages were. For example, *Amphinema byssoides*, *Cenococcum geophilum*, and *Cortinarius scaurus* and *Piloderma byssinum* all had relatively high amide/protein content in relation to the polysaccharide, whereas *Piceirhiza bicolorata* and *Suillus bovinus* had a far greater proportion of polysaccharide relative to amide/protein (Fig. 2). *Piceirhiza bicolorata* also had very intense absorption bands consistent with an unsaturated waxy ester compound (Fig. 2b), which the spectra of the other species did not show.

**Fig. 3:**
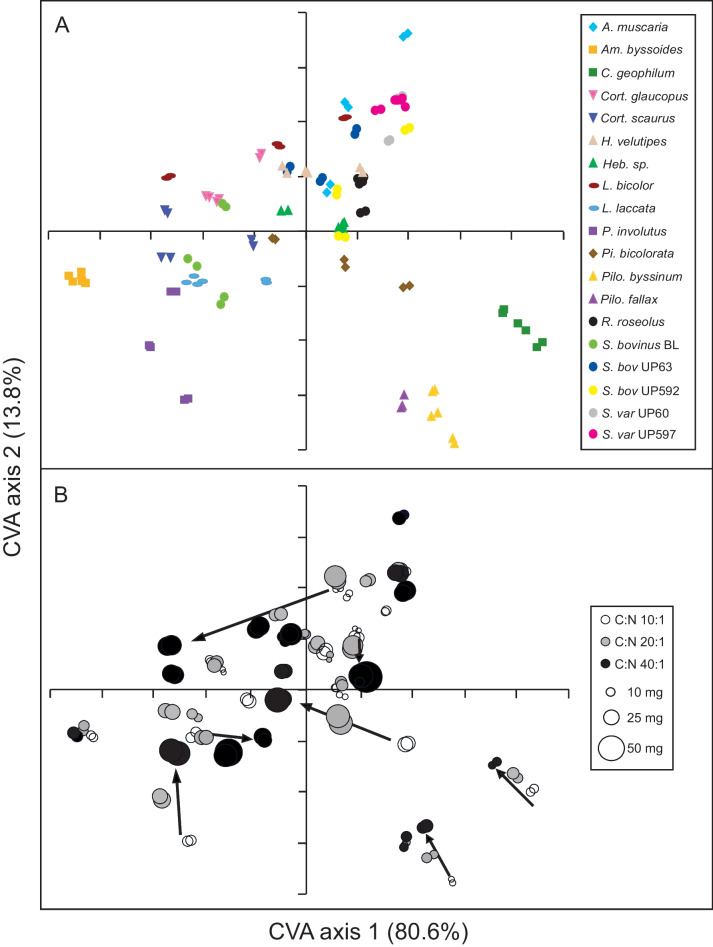
Large species-specific differences in chemical composition, and varied responses to increased C availability. Canonical variate analysis of FTIR spectra for ECM fungal isolates grown for 3 weeks in liquid media at three different C:N ratios (10:1, 20:1, and 40:1) with an inorganic N source ((NH_4_)_2_SO_4_), showing the difference between (**a**) fungal species, and (**b**) C:N ratios. For each treatment, there were two replicates. Biomass for each individual sample is indicated by the size of the circle in **b**, and arrows exemplify the direction of change in chemical composition when a species experienced increasing C availability

Differences in chemical composition among the 19 isolates were visualized in the CVA (Fig. 3a), where the first three CVA axes together accounted for 98% of the variation. The adjusted loadings for the first two variates showing which regions of the FTIR spectra are important for separating samples in multivariate space are shown in Fig. S2. There were strong and significant effects of ECM species (perMANOVA *F* = 266.2, *R*^2^ = 0.873, and *P* = 0.001; Table 2) and large species-specific differences in chemical composition (Fig. 3a). Species were generally separated from each other in multivariate space within a single C:N ratio treatment (Fig. S4), with few exceptions. In C:N ratio 10:1 *Hebeloma* sp. 1 and *S. bovinus* UP592, *Hebeloma velutipes* and *R. roseolus* overlapped with each other, and in C:N ratio 40:1 *H. velutipes* and *S. bovinus* UP63 overlapped. The two isolates of *S. variegatus* (UP597 and UP60) were similar in chemical composition in C:N ratio 40:1. The spread among species within the individual treatments was similar irrespective of C availability (Fig. S4). The separation of species in multivariate space was therefore primarily explained by species’ differences. However, when taking all three C:N ratios into account at the same time species start overlapping (Fig. 3a), and only some species (e.g., *C. geophilum*, *A. byssoides*, and *Paxillus involutus*) remained clearly separated from all the other species. Closely related species pairs such as the two *Piloderma* spp., the two *Cortinarius* spp., and the two *Hebeloma* isolates grouped relatively closely together within genus in the ordination (Fig. 3a), and the *Piloderma* spp. were also clearly separated as a group from all other species. The similarity within these genera was also obvious from the IR spectra (not shown). *Laccaria bicolor* and *L. laccata*, on the other hand, showed a large spread from each other in the multivariate analysis, also within separate C:N treatments (Fig. S4). This separation was possibly explained by differences in relative proportions of chitin. The *L. laccata* spectra show absorption bands in the polysaccharide region (1200–900 cm^−1^) which have a pattern very similar to that of chitin and also show a relatively strong band at 1311 cm^−1^, which is also present in the spectrum of chitin. Conversely the *L. bicolor* spectra have a different pattern of bands in this region (more like reference spectra of isomaltose, possibly more glucan like). The suilloid isolates (6 isolates corresponding to three species: *S. bovinus*, *S. variegatus*, and *R. roseolus*) grouped together (however fully overlapping with other species) with the exception of *S. bovinus* BL that differed in chemical composition (Fig. 3). This was mainly due to the presence of an aromatic peak and less distinct polysaccharide backbones in *S. bovinus* BL but not in the other suilloid isolates (Fig. S5).

**Table 2:** Variation in chemical composition based on FTIR spectra (perMANOVA analysis) in relation to ECM species and C-availability for 17 ECM isolates grown in pure culture at C:N ratios 10:1, 20:1, and 40:1, testing overall effects of (a) ECM species and C:N ratio, and (b) ECM species, C:N ratio, and biomass. Values of degrees of freedom (DF) and sums of squares (SS) are shown

	DF	SS	*R*^2^	F	Pr (> F)
(a)					
Species	16	21,278.0	0.87283	266.224	0.001
C:N ratio	2	239.0	0.00980	23.922	0.001
Species * C:N ratio	32	2606.4	0.10691	16.305	0.001
Residual	51	254.8	0.01045		
Total	101	24,378.2	1.00000		
(b)					
Species	16	21,278.0	0.87283	265.2366	0.001
C:N ratio	2	239.0	0.00980	23.8333	0.001
Biomass	1	50.0	0.00205	9.9744	0.001
Species * C:N ratio	32	2560.4	0.10503	15.9582	0.001
Residual	50	250.7			
Total	101	24,378.2			

### Effects of increasing C-availability on chemical composition

**Fig. 4:**
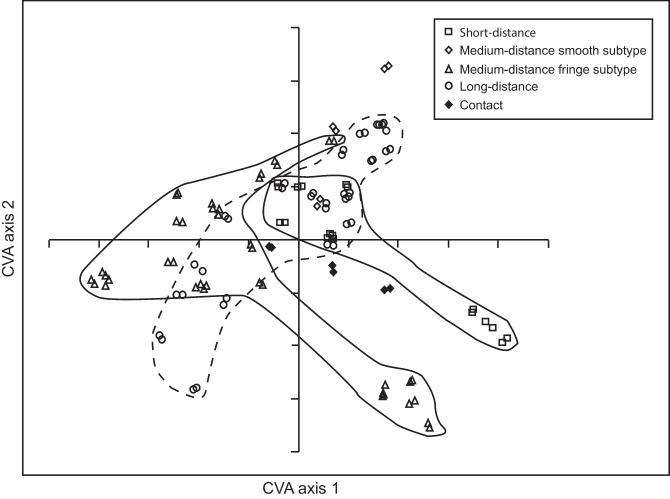
Separation in multivariate space was significantly explained by exploration types (MRPP *A* = 0.2034, *P* = 0.001), although the various exploration types partly overlap in terms of chemical composition. Canonical variate analysis of FTIR spectra for ECM fungal isolates grown at three different C:N ratios (10:1, 20:1, and 40:1; same CVA as Fig. 3). ECM species were classified according to their pattern of mycelial differentiation and putative ecological role (Agerer 2001)

FTIR data showed a significant effect of C:N ratio on chemical composition, but C:N ratio explained a much smaller part of the variation compared to ECM species (perMANOVA *F* = 23.9, *R*^2^ = 0.0098, and *P* = 0.001; Table 2). However, there was also a significant interaction between ECM species and C:N ratio explaining 11% of the variation in chemical composition (perMANOVA *F* = 16.3, *R*^2^ = 0. 107, and *P* = 0.001; Table 2). The multivariate analysis showed varied responses in terms of chemical composition among different species, and also among isolates of the same species, to increasing C:N ratio (Fig. 3b). The responses ranged from no shift in chemical composition irrespective of C availability (*A. byssoides* and *P. fallax*; Fig. 3a), to large differences in composition so that the C:N ratio treatments were completely separated (e.g., *P. bicolorata*, *L. bicolor*, and *A. muscaria*; Fig. 3a). Separation in multivariate space was significantly explained by exploration type (MRPP *A* = 0.2034, *P* = 0.001; Fig. 4), hydrophobicity (MRPP *A* = 0.112, *P* = 0.001; Fig. S6a), N tolerance (MRPP *A* = 0.08632, *P* = 0.001; Fig. S6b), and succession (MRPP *A* = 0.05828, *P* = 0.001; Fig. S6c) when ECM fungal species were classified according to these traits (see classifications in Table 1). When classifying ECM species according to their pattern of mycelial differentiation and putative ecological roles, the chemical composition of short-distance exploration types was mostly separated from medium-distance fringe subtypes, while long-distance exploration types partly overlapped with both former groups (Fig. 4).

**Fig. 5:**
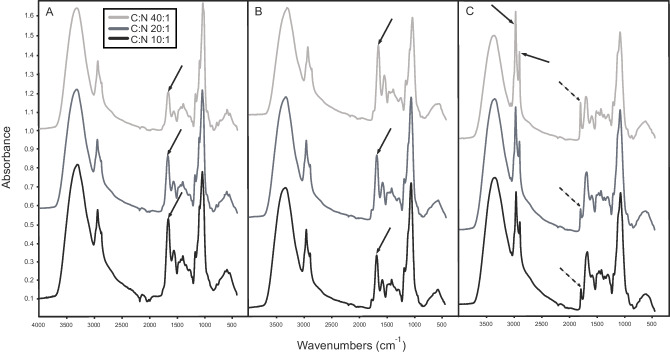
Changed C availability alters chemical composition of ECM fungi differently depending on species. FTIR spectra for (**a**) *A. muscaria*, (**b**) *L. bicolor*, and (**c**) *P. bicolorata* mycelia grown at three different C:N ratios with an inorganic N source ((NH_4_)_2_SO_4_). Arrows in **a** indicate reductions of amide peaks with increasing C:N ratio for *A. muscaria*, representing the most obvious change in chemical composition in response to increased C availability. In **b**, the arrows indicate the amide peak which was highest with C:N ratio 40:1, and *L. bicolor* represents a group of species together with *P. bicolorata* that showed increasing relative proportions of amide with increasing C availability. In **C**, black arrows indicate large CH_2_ peaks and the dashed arrows indicate the ester peak for *P. bicolorata*, showing subtle changes in waxy character in the C:N ratio 10:1. Absorbance (spectral signals) was normalized to give relative absorbance/abundance, and spectra are shown off set

The IR spectra for different species varied markedly in response to different C:N ratios, and four groups of responses could be distinguished. Firstly, many of the most obvious changes were related to the relative proportions of the amide (or protein) bands and the polysaccharide bands, with a clear reduction in amide with increasing C availability (e.g., *A. muscaria*; Fig. 5a; see Fig. S7 for the same spectra plotted overlaid). *Amanita muscaria* also had very distinct ring linkages (1074, 572, and 528 cm^−1^). The pattern with increasing polysaccharide bands was also similar but less pronounced for *P. bicolorata* (Fig. 5c), and *C. geophilum*, *Cortinarius glaucopus*, *L. laccata*, *P. byssinum*, *S. bovinus* UP592, and *S. variegatus* UP60 (not shown). *Piloderma byssinum* had intense amide peaks, and possible contribution from a carboxylate band in the amide region (Fig. 2a). Secondly, one group moves in the opposite direction with increasing relative proportions of amide with increasing C availability (e.g., *L. bicolor*; Fig. 5b; *S. bovinus* UP63; Fig. S5). *Laccaria bicolor* showed subtle changes in waxy character with most CH_2_ and ester at C:N ratio 10:1 (Fig. 5b), and *P. bicolorata* had a strong waxy character with very sharp CH_2_ bands and evidence for an acid, in addition to ester, in C:N 20:1 (Fig. 5c). As a proxy for how mycelial C:N ratio was affected by C availability for *A. muscaria* and *L. bicolor*, representing the two first groups of responses mentioned above, the relative height of the main polysaccharide peak was compared to that of the amide peaks (Table 3). This showed a decreased amount of protein for *A. muscaria* (ratio increasing from 1.7 to 3.4), and increased amount of protein for *L. bicolor* (ratio decreasing from 2.1 to 1.2), respectively, with increased C availability. Thirdly, one group (*A. byssoides*, *P. fallax*, and *S. variegatus* UP597) showed little change in response to C availability (not shown). *Amphinema byssoides* had some of the highest amide peaks of all species (Fig. 2a), and *P. fallax* had an oxalate peak (Fig. S8; 1317 cm^−1^). Fourthly, the remaining species showed a mixture of responses not including the amide/polysaccharide response. *Cortinarius scaurus* had intense amide bands in all treatments (Fig. 2a, C:N ratio 20:1) and greater CH_2_ intensity at C:N ratio 10:1. *Hebeloma velutipes* had slightly greater CH_2_ intensity but overall little change in pattern, and *Hebeloma* sp. 1 showed some more aromatic character at C:N ratio 40:1 (not shown). Furthermore, *P. involutus* had an obvious aromatic peak at 1515 cm^−1^ (see Fig. S5 for the aromatic peak in *S. bovinus*), and ring linkages (1074, 572, and 528 cm^−1^). Finally, *R. roseolus* showed no changes in amide bands but a large change in waxy ester bands especially at C:N ratio 40:1 (Fig. S1), and *S. bovinus* BL had a distinct aromatic component which decreased at low C availability (Fig. S5). In addition to these four groups of responses to increased C availability, there were also more subtle changes in the nature of the polysaccharide noted in some cases, in particular how distinct the bands relating to ring linkages changed with C:N ratio.

**Table 3:** Comparison between the relative height of the main C-O stretching vibration (~ 1030 cm − 1, associated with polysaccharides) and the relative height of the amide bands (amide I ~ 1650, amide II ~ 1550 cm − 1, associated with N) for *Amanita muscaria* and *Laccaria bicolor*, used as a proxy for mycelial C:N ratio. Relative height is given corrected for baseline. Uncorrected peak height in ()

Species	C:N ratio	C-O peak height (a)	Amide 1 peak height (b)	Amide 2 peak height (c)	Ratio a/(b + c)
*A. muscaria*	10:1	0.405 (0.449)	0.183 (0.288)	0.057 (0.177)	1.688
	20:1	0.526 (0.591)	0.182 (0.298)	0.055 (0.185)	2.219
	40:1	0.619 (0.663)	0.143 (0.217)	0.037 (0.121)	3.439
*L. bicolor*	10:1	0.511 (0.591)	0.18 (0.296)	0.059 (0.186)	2.138
	20:1	0.468 (0.514)	0.203(0.290)	0.076 (0.178)	1.677
	40:1	0.266 (0.326)	0.158 (0.248)	0.065 (0.166)	1.193

### Mycelial biomass

Although chemical composition was significant influenced by biomass, the variance explained was very small (perMANOVA *F* = 9.97, *R*^2^ = 0.002, and *P* = 0.001). Biomass dry weight for each sample is depicted in the sample plot from the multivariate analysis (Fig. 3b), ranged from ca. 3 to 27 mg, 5 to 49 mg, and 11 to 60 mg at C:N ratios 10:1, 20:1, and 40:1, respectively (Table S2), and was significantly affected by both fungal species (two-way ANOVA *P* < 0.001) and C availability (two-way ANOVA *P* < 0.001). In addition, there was a significant interaction effect (two-way ANOVA fungal species × C availability *P* < 0.001). Most of the tested isolates (13) increased biomass with increasing C availability (producing the smallest biomass at C:N ratio 10:1 and the largest at C:N ratio 40:1), while two isolates (*P. fallax* and *S. bovinus* UP592) produced very similar amount of biomass at C:N ratios 10:1 and 20:1 and one isolate (*S. variegatus* UP60) produced similar biomass at C:N ratios 20:1 and 40:1. Three species (*A. muscaria*, *C. geophilum*, and *P. bicolorata*) produced maximum biomass at intermediate C availability (C:N ratio 20:1). Isolates with the largest increase in biomass at C:N ratio 40:1 compared to 10:1 also exhibit large differences in chemical composition, e.g., *S. bovinus* BL, UP63 and UP592, *L. bicolor*, and *H. velutipes* (Fig. 3), although not exclusively so (e.g., *A. muscaria* decrease in biomass at C:N ratio 40:1 compared to 10:1 but still show large differences). The two ascomycetous isolates (*C. geophilum* and *P. bicolor*) showed large differences in chemical composition when comparing C:N ratio 40:1 and 10:1 despite small increases in biomass (Fig. 3). Finally, there were some isolates with smaller biomass increases at C:N ratio 40:1 compared to 10:1 that also showed small differences in chemical composition, e.g., *A. byssoides* and *P. fallax* (Fig. 3).

## Discussion

Carbon availability has been suggested to be the key factor determining mycelial production and possibly also its standing biomass in boreal and temperate forests, and any factors regulating C availability from the host plant such as global change, forest age (Hagenbo et al. 2017), forestry management, and plant properties as well as fungal C use (Hagenbo et al. 2019) can potentially cause large variations in mycelial production of ECM fungi (Ekblad et al. 2013). In this study, we tested the hypothesis that C availability impacts not only on the production but also directly on the chemical composition of mycelia, and we expected an increased C availability to lead to more carbohydrates and less proteins. We showed that C availability significantly impacted chemical composition, and for many species, the levels of carbohydrates increased while proteins decreased. However, this was only part of the pattern since different species varied markedly in response to C:N ratios, and ECM species explained by far the largest part of the variation in IR spectra.

### Large species-specific differences in chemical composition

When C availability increased, more than 70% of the tested isolates responded with increased biomass production, although the magnitude differed depending on species and/or isolate. This biomass increase was in most cases associated with differences in chemical composition, confirming our overall hypothesis that C availability impacts both production (Ekblad et al. 2013) and chemical composition of mycelia. The 19 ECM isolates showed large species-specific variation in chemical composition (and mycelial production) and in their response to C availability as was evident from the significant interaction between ECM species and C:N ratio (perMANOVA), and species could mostly be distinguished from each other under the same growth conditions (C:N ratio), by using FTIR fingerprinting. Ectomycorrhizal fungi typically show large species-specific variation in growth and function (e.g., Abuzinidah and Read 1986; Agerer 2001; Cairney 1999; Koide and Malcolm 2009), but also intraspecific variation may be important (Hazard et al. 2017). Changes in the utilization of C and N among ECM fungi have been reported from many studies, with increased mycelial biomass at higher C availability (but depending on total C and N levels) in pure culture (e.g., Alexander 1983; Baar et al. 1997; Fransson et al. 2007; Itoo and Reshi 2014) and pots and field experiments (CO_2_ meta-analysis; Alberton et al. 2005). Variation in chemical composition (beyond C and N content) among ECM species is less well studied. Species-specific patterns have been reported for ECM fungi in pure culture, symbiosis, and fruitbodies, for example, whole cell fatty acid composition (Karliński et al. 2007), fungal soluble carbohydrate concentrations (Koide et al. 2000), and N, starch, and soluble sugar concentrations (Trocha et al. 2010). For ECM, mycelia chemical composition has mostly received attention in the context of decomposition and soil organic matter sequestration. Species-specific differences in chemical composition in this context were reported for melanization (Fernandez and Koide 2014; *Meliniomyces bicolor* intra-specific variation: Fernandez and Kennedy 2018), N concentration (Koide and Malcolm 2009), and chitin (Fernandez and Koide 2012). Fernandez and colleagues (2016) concluded that the C:N ratio of ECM necromass is an important factor governing decomposition, but that its role is modulated by the nature of the C and N in the mycelia. Finally, Yang and colleagues (2019) investigated how leaf litter quality influenced the biochemical profiles of mycorrhizal root tips by planting young beech trees in an oak forest and replacing the natural leaf litter with other trees species’ litter. They found that chemical composition changed with leaf litter species in a species-specific manner, with apparent changes in the infrared absorption bands assigned to functional groups of lipids, amides, and carbohydrates, similarly to the present study. Yang et al. (2019) suggested that the biochemical composition of ECM mycelium is a fungal response trait, sensitive to environmental changes. These findings together point to the importance of understanding mycelial chemical composition, and how it varies among species and under different conditions.

### Species variation in chemical composition related to structural components

The predominant compounds identified by FTIR were polysaccharides and amide (proteins), which is in line with what is previously known about fungal cell wall composition (Bartnicki-Garcia 1968; Fernandez et al. 2016). What some of the other constituents responsible for differences in chemical composition among ECM species may be related to structurally was sometimes not clear. The waxy compounds correspond to long chain aliphatic hydrocarbons and could either be CH-based only, or attached to ester or acid functional groups. The unsaturated waxy esters in the ascomycete *P. bicolorata* were unique to this species, but we do not know the function. Of the six closely related suilloid isolates, all but one shared similar cell wall composition; the presence of an aromatic peak in *S. bovinus* BL may correspond to melanin or other similar compounds, separating this isolate from the others in multivariate space.

Melanins, a group of complex polymers of phenolic or indolic monomers forming negatively charged, hydrophobic pigments of high molecular weight (White 1958), result in the black or brown color of, e.g., *C. geophilum* and *P. bicolorata*. Ascomycetes produce different types of melanin compared to basidiomycetes, and in cell walls, melanin is likely cross-linked to polysaccharides (Butler and Day 1998). Melanins are important for protection and stress tolerance, and the recalcitrance and decomposition of fungal mycelia (Fernandez and Koide 2014). An observed difference in aromatic compounds in *C. geophilum* mycorrhizal root tips grown with beech litter compared to other leaf litter species in a field experiment may have been explained by a higher melanin content (Yang et al. 2019). Furthermore, melanin correlated negatively with fungal biomass production across 62 fungal species (Siletti et al. 2017), suggesting a physiological trade-off. The isolates we tested, however, did not follow this pattern clearly since *P. bicolorata* produced relatively large biomass. Chitin is usually complexed with other compounds such as glucans, proteins, and melanins (Bartnicki-Garcia 1968), but could be detected in, e.g., *L. laccata*. Chitin is used as an indicator of all fungal biomass integrated over the lifespan (Ekblad et al. 1998). Similar to melanin, chitin has been suggested to affect decomposition of mycelia, but Fernandez and Koide (2012) showed that chitin was not recalcitrant relative to other compounds in fungal tissues, and that its concentration was positively related to the decomposition. Unlike other structural polysaccharides, chitin contains substantial concentrations of N, likely contributing to the decomposability.

The oxalate peak in *P. fallax* showed an oxalate presence. This species produces ornamented hyphae that are coated with crystals of calcium oxalate or other crystalline deposits (Brand 1991a, b; Taylor and Alexander 2005), hypothesized to make them unattractive as food for fungal grazers such as mites. Exudation of oxalate, the most commonly produced organic acid, varies significantly between ECM species and responds to, e.g., increased C availability (Johansson et al. 2009), and plays important roles for biological mineral weathering and in formation of secondary minerals via the oxalate–carbonate pathway (Finlay et al. 2020). Organic acids and other exudates such as amino acids, sugars, and pigments that are water soluble should however mostly not impact the chemical composition of mycelia when they are continuously released into the surrounding soil solution. Chitin is usually complexed with other compounds such as glucans, proteins, and melanins (Bartnicki-Garcia 1968), but could be detected in e.g., *L. laccata*. Changes in ECM community composition and species variation in chemical composition may ultimately lead to changes in soil C sequestration if species with a higher proportion of recalcitrant compounds such as melanin become more common in response to changed environmental conditions.

### Large differences in the type of response to increased C-availability among ECM fungal species

Carbon availability directly and significantly impacted the chemical composition of ECM mycelia, but not for all tested species. The expected increase in carbohydrates and reduction in amide (less proteins) in the cell walls was observed for half of the isolates. The two other types of strategies in response to increased C availability included increasing relative proportions of amide, which is the exact opposite to what we expected, and no change. This revealed that responses to C availability among ECM species are not general, instead changes in C utilization are complex. Even when looking at closely related species, for example, the suilloid fungi, all three main responses were present among the six tested isolates in this group. Suilloid species are known to be C demanding (Kuikka et al. 2003), produce abundant extraradical mycelia and extensive rhizomorph systems (Agerer 2001), and were highly responsive to increasing C availability in terms of biomass production and respiration (Fransson et al. 2007). We expected this group to behave in a similar way, increase in carbohydrates and decrease in proteins with high C availability, but instead they displayed increased polysaccharide bands together with decreased amide, increased relative proportions of amides, or little change in chemical composition. It should, however, be noted that the nature of the amide/protein material does vary from species to species—differences in the amide I and II position and shape can be indicative of different protein structures or may relate to different amide containing material. Furthermore, different responses to C availability may reflect ecological differences between groups or species, and we could relate both mycelial differentiation type (exploration type), and putative ecological roles such as N tolerance, hydrophobicity, and succession significantly to chemical composition in the present study. Exploration types, a functional trait used to explore, e.g., nutrient acquisition, have also been proposed to be linked to fungal C demand and host photosynthetic capacity (Defrenne et al. 2019; Köhler et al. 2018; Wasyliw and Karst 2020). How exploration types or other functional traits relate to chemical composition of mycorrhizal fungi would need to be explored further in detail to elucidate possible patterns and to understand the differences among ECM species.

### Usefulness of the FTIR method to distinguish ECM species and explain differences in chemical composition

Despite the fact that we analyzed only two replicates per treatment, reproducibility of fungal FTIR spectra was generally high. The replicate spectra could be almost exactly overlaid which indicates that the chemical compositions were nearly identical, and the coefficients of variation were low for the majority of treatments. Differences between spectra could therefore be reliably taken to show differences in chemical composition for the fungal mycelium growing under different conditions. Interpretation of the FTIR spectra for all the species in this study has provided a wealth of information on the variation in chemical composition. We determined that FTIR spectroscopy can be used to distinguish between different ectomycorrhizal species under the current set up, especially when they were grown under the same growth conditions, and that species explained as much as 87% of the variation in chemical composition. Since the experiment was done under highly controlled conditions, the ecological relevance of our finding may be questioned since the fungal performance together with its host interacting with the environment may be quite different. Due to the very limited knowledge about the chemical composition of ECM mycelia, investigating the potential differences that ECM species can display is still valuable. The use of in situ collected mycorrhizal root material by Pena et al. (2014) showed that four distinctly different morphotypes could be readily distinguished from each other; however, there are issues with recording spectra of in situ spectra as mineral material present will significantly alter the FTIR spectrum. How well our results correspond to differences in field collected extraradical mycelia grown under different conditions, and whether the differences in chemical composition may be as large and useful for distinguishing a variety of ECM fungal species remains to be tested.

## Conclusions

Fernandez and colleagues (2016) concluded that the C:N ratio of ECM necromass is an important factor governing decomposition, but that its role is modulated by the nature of the C and N in the mycelia. Variation in chemical composition among ECM species is, however, not well understood. To understand the decomposition process of mycelia, we need more studies on how growth conditions in terms of C or nutrient availability may impact on the chemical composition of mycorrhizal mycelia in the soil, and how this couples to the ECM community composition. We showed that C availability impacted significantly on the chemical composition of mycelia, and we conclude that ECM species explained the major part of the variation in chemical composition and can mostly be discriminated from each other by FTIR, especially when compared within the same treatment using a pure culture setup. Given that the responses of different species (and isolates) varied markedly in response to C:N ratios and that the species variation in chemical composition related to different types of structural components, changes in community composition may ultimately lead to changes in soil C sequestration if species with a higher proportion of recalcitrant compounds such as melanin become more common in response to changed environmental conditions. The approach of using FTIR looks promising and further species could be tested under a wider set of environmental and physiological controls to further elucidate linkages to their ecological role.

### Supplementary Information

Below is the link to the electronic supplementary material.

Supplementary file1 (PDF 4090 KB)

Supplementary file2 (PDF 1647 KB)

## Data Availability

FTIR raw data are available in Dryad (Fransson et al. 2023).
