# Supplementary material for: Carbon availability affects already large species-specific differences in chemical composition of ectomycorrhizal fungal mycelia in pure culture
Source: Mycorrhiza. 2023 Oct 12;33(5-6):303–19. doi: 10.1007/s00572-023-01128-2 (PMC10752919; doi:10.1007/s00572-023-01128-2)
Supplement: Supplementary file 1 — Supplementary file1 (PDF 4090 KB) [file 572_2023_1128_MOESM1_ESM.pdf]

**Carbon availability affects already large species-specific differences in chemical composition of ectomycorrhizal fungal mycelia in pure culture**

Mycorrhiza

Petra Fransson[1*], A.H. Jean Robertson[2] and Colin D. Campbell[2]

[1] Uppsala BioCenter, Department of Forest Mycology and Plant Pathology, Swedish University of Agricultural Sciences, PO Box 7026, SE-75007 Uppsala, Sweden

[2] The James Hutton Institute, Craigiebuckler, Aberdeen AB15 8QH, Scotland

*** Correspondence:**

Petra Fransson e-mail: petra.fransson@slu.se

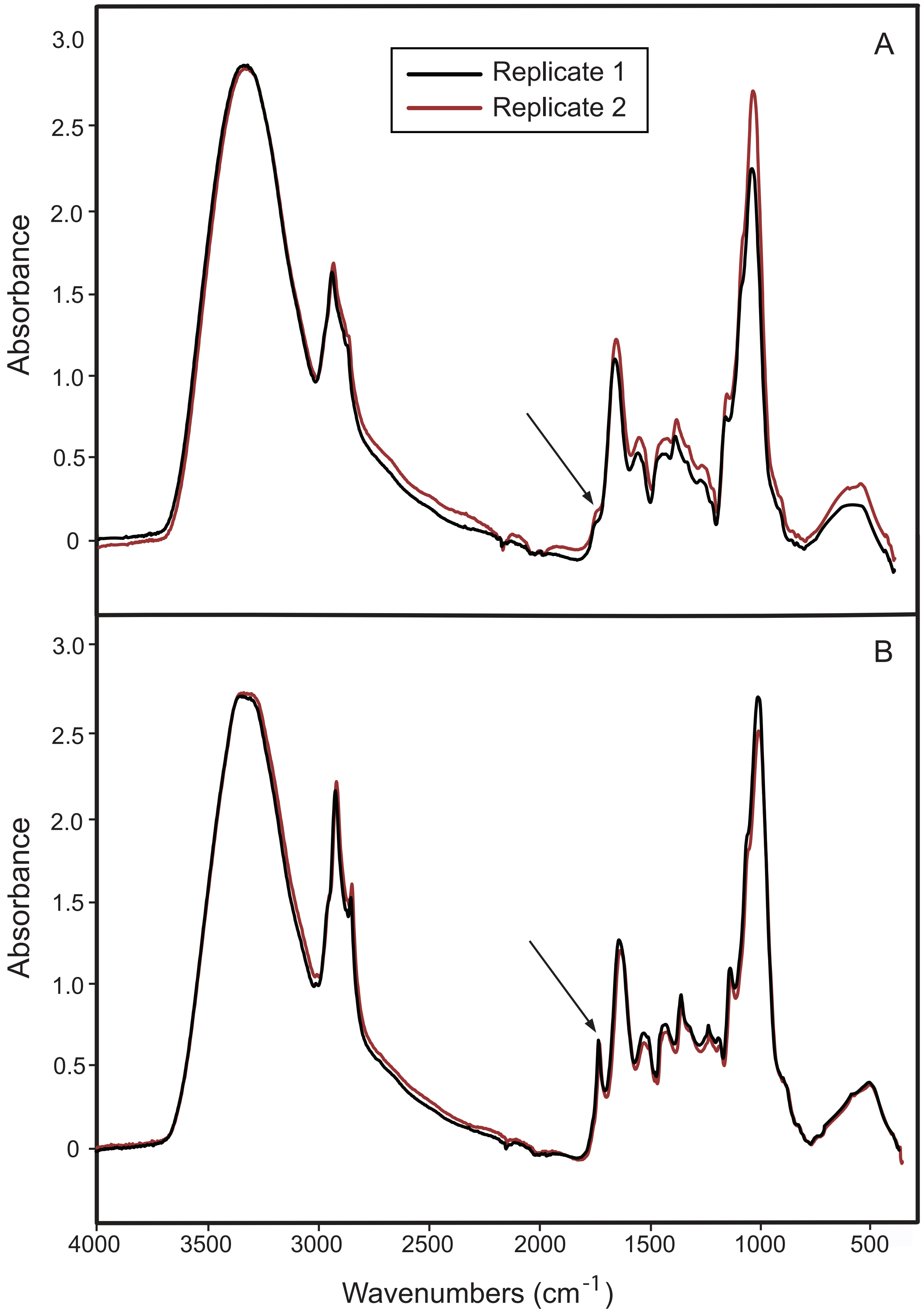


**Supplementary Fig. 1.** Replicates within treatments showed high consistency in chemical composition (evaluated by average coefficients of variation, see Table S1). FTIR spectra for *R. roseolus* mycelia, grown either at C:N ratio (A) 10:1 or (B) 40:1. The arrows shows the large change in waxy esters with increasing C availability. Absorbance (spectral signals) was normalised by subtraction of the minimum value and subsequent division by the average of all data points per sample prior to statistical analyses to give relative absorbance/abundance.

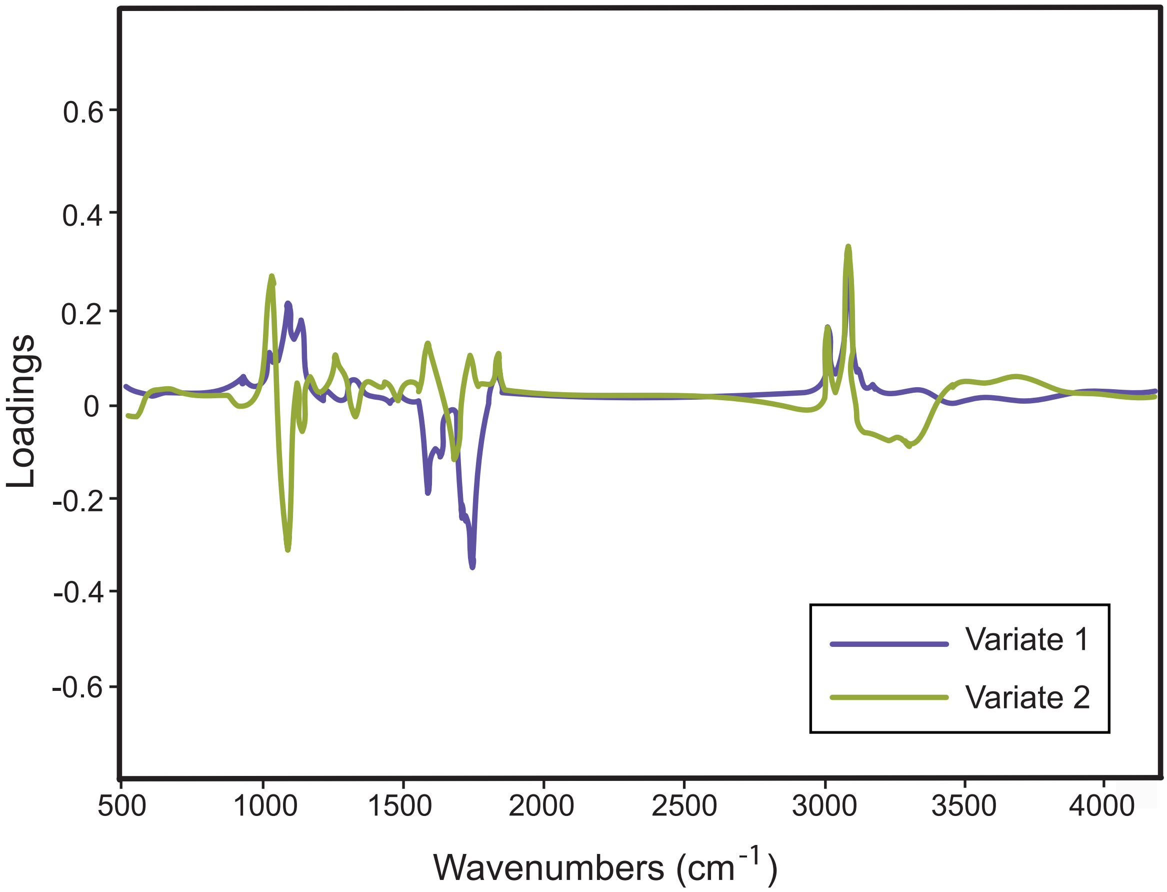


**Supplementary Fig. 2.** The adusted loadings (or coefficients of linear discriminants for each absorbance value) for the original variates (variates 1 and 2) are plotted, showing which regions of the FTIR spectra are important for separating samples in multivariate space. Canonical variate analysis (CVA) proceeded by principal components analysis (PCA) was used to analyse ECM fungal FTIR spectra. Treatments included 15 different species and a total of 19 isolates grown at three C:N ratios (10:1, 20:1 and 40:1) simulating increasing carbon availability.

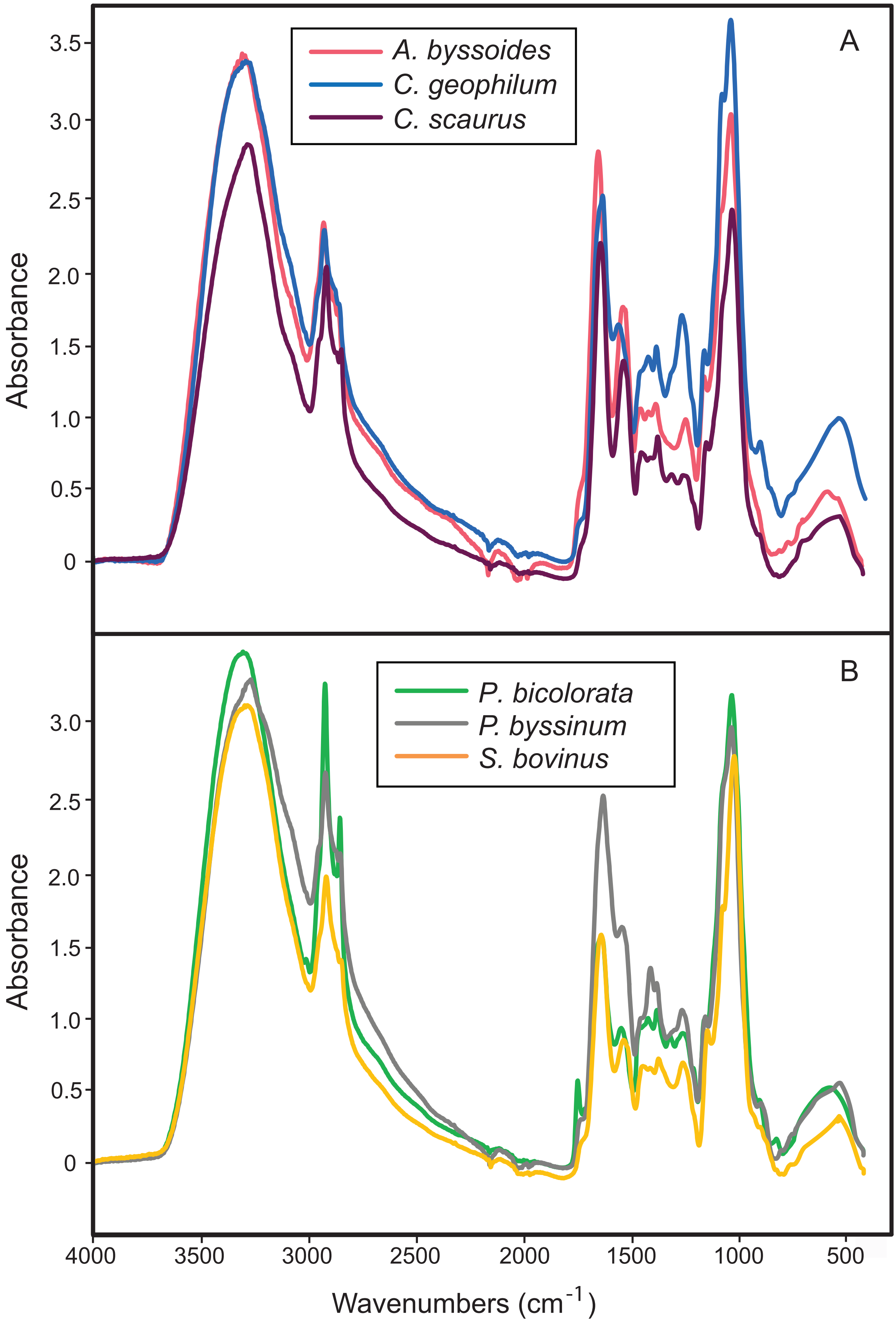


**Supplementary Fig. 3.** Species variation in FTIR spectra for different ECM fungal species, showing (A) *A. byssoides*, *C. geophilum* and *C. scarus*, and (B) *P. bicolorata*, *P. byssinum* and *S. bovinus*. Mycelia were grown in pure culture at C:N ratio 20:1 with an inorganic nitrogen source ($(NH_4)_2SO_4$). Absorbance (spectral signals) was normalised to give relative absorbance/abundance. Same data as shown off set in Fig. 2.

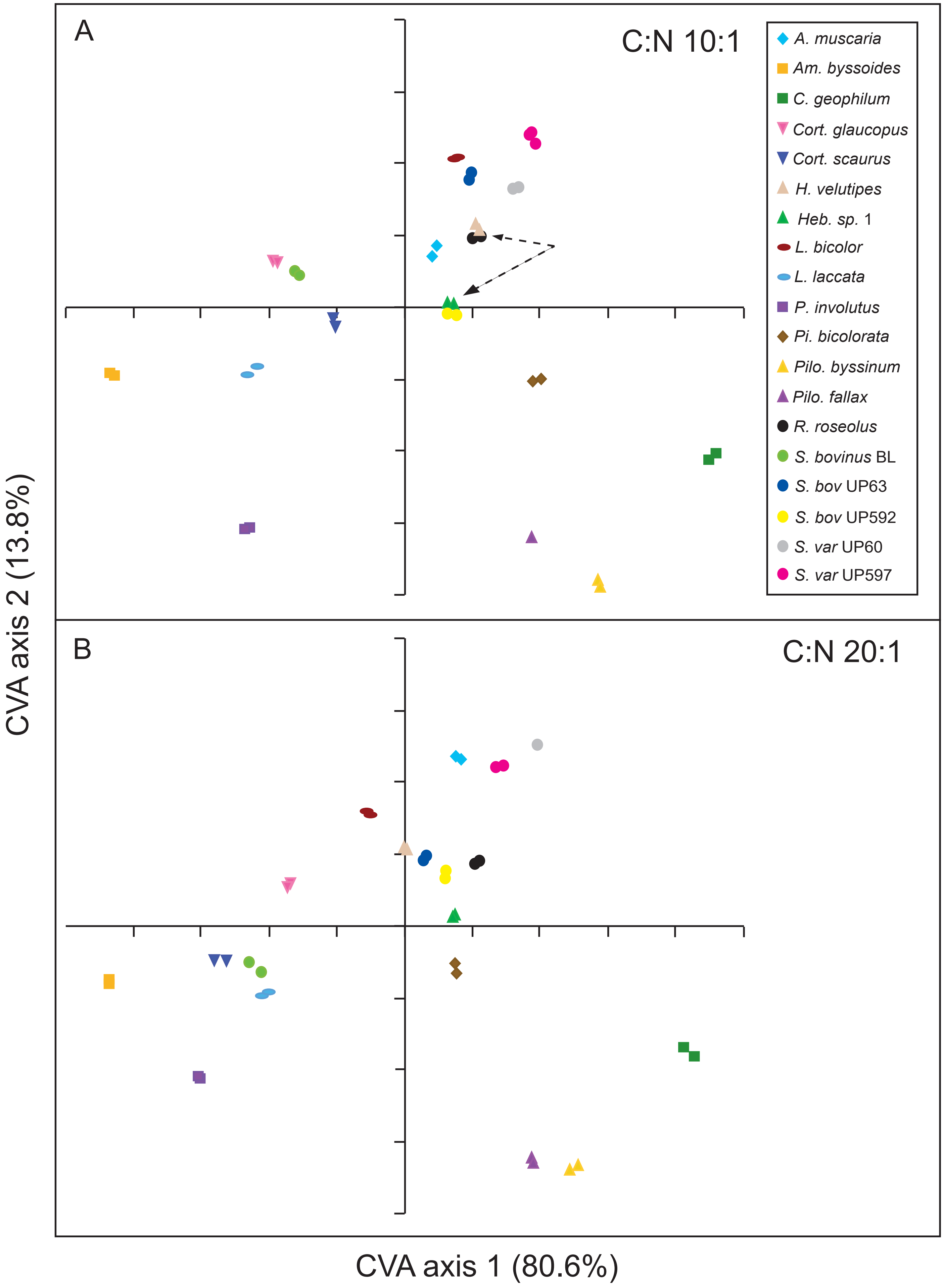
A
C:N 10:1
A. muscaria
Am. byssoides
C. geophilum
Cort. glaucopus
Cort. scaurus
H. velutipes
Heb. sp. 1
L. bicolor
L. laccata
P. involutus
Pi. bicolorata
Pilo. byssinum
Pilo. fallax
R. roseolus
S. bovinus BL
S. bov UP63
S. bov UP592
S. var UP60
S. var UP597
B
C:N 20:1
CVA axis 2 (13.8%)
CVA axis 1 (80.6%)

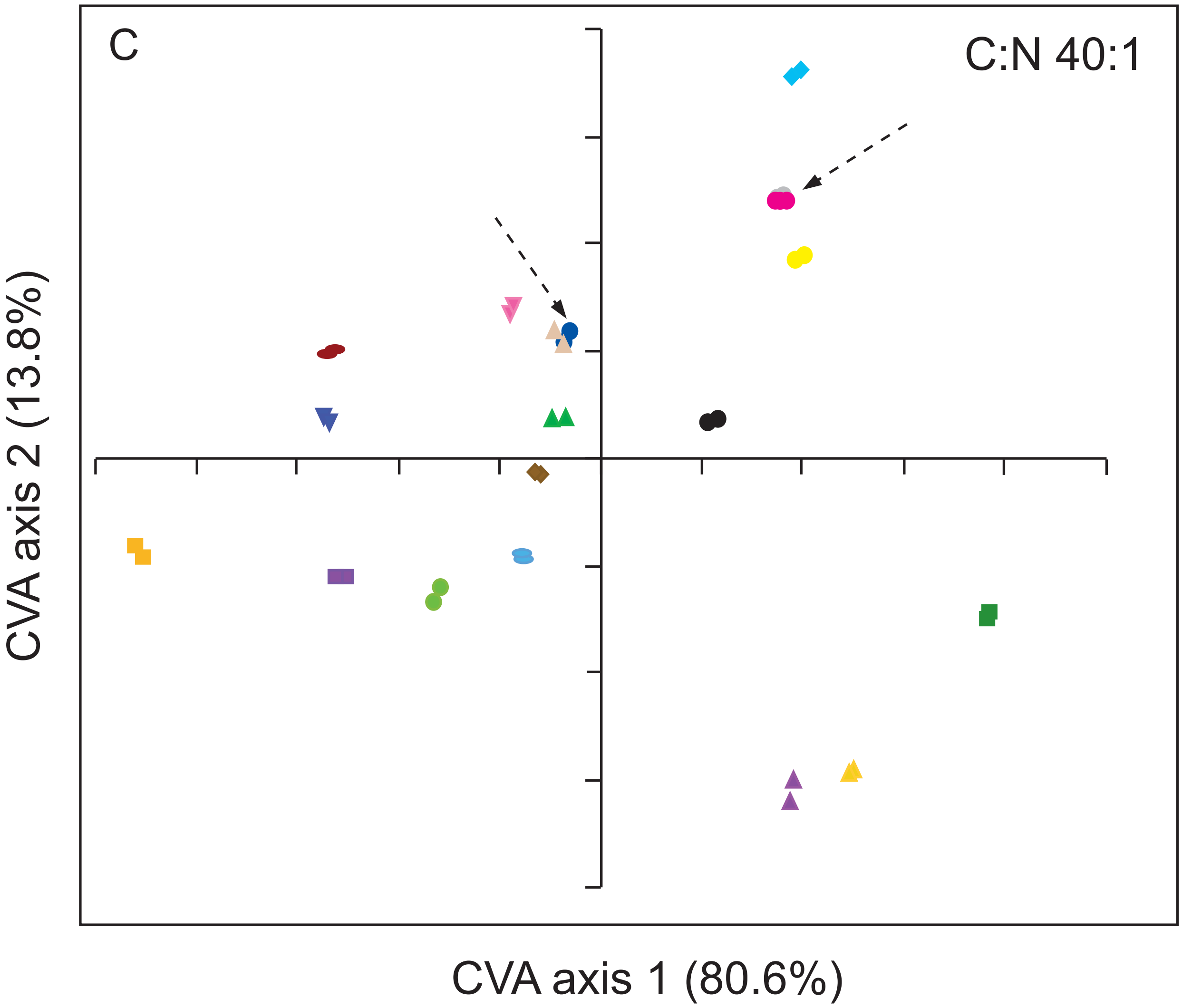


**Supplementary Fig. 4.** Different ECM fungal species can generally be differentiated from eachother irrespective of C availability. Canonical variate analysis of FTIR spectra for 19 ECM fungal isolates grown for three weeks in liquid media at C:N ratios (A) 10:1, (B) 20:1, and (C) 40:1. Dashed arrows show overlapping species with similar chemical composition within a C:N ratio. For each treatment there were two replicates. Same analysis and results as reported in Fig. 3, showing one C:N treatment at the time.

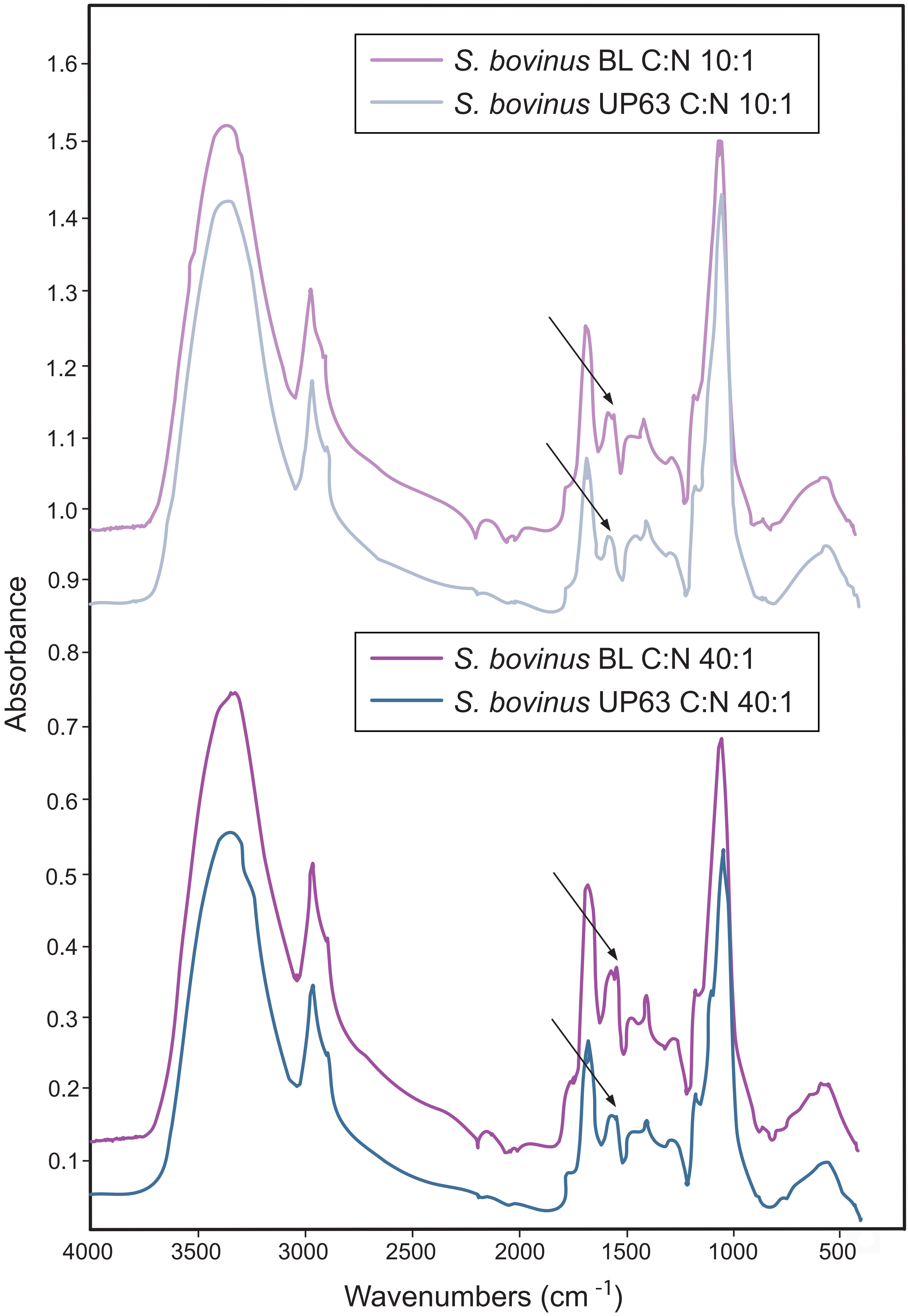

S. bovinus BL C:N 10:1
S. bovinus UP63 C:N 10:1
S. bovinus BL C:N 40:1
S. bovinus UP63 C:N 40:1
Absorbance
Wavenumbers (cm -1)
1.6
1.5
1.4
1.3
1.2
1.1
1.0
0.9
0.8
0.7
0.6
0.5
0.4
0.3
0.2
0.1
4000
3500
3000
2500
2000
1500
1000
500

**Supplementary Fig. 5.** Carbon availability has different effects on the chemical composition of isolates of the same ECM fungal species. FTIR spectra for two isolates of *S. bovinus* (isolates BL and UP 63) grown either at low C:N ratio (10:1) or high C:N ratio (40:1) with an inorganic nitrogen source ($(NH_4)_2SO_4$). Arrows indicate a small aromatic peak at 1515 $cm^{-1}$ in isolate BL at low carbon availability, that is not evident in isolate UP63 at the same carbon availability. At high carbon availability this peak is stronger for BL and emerging in the spectrum for UP63. Absorbance (spectral signals) was normalised to give relative absorbance/abundance.

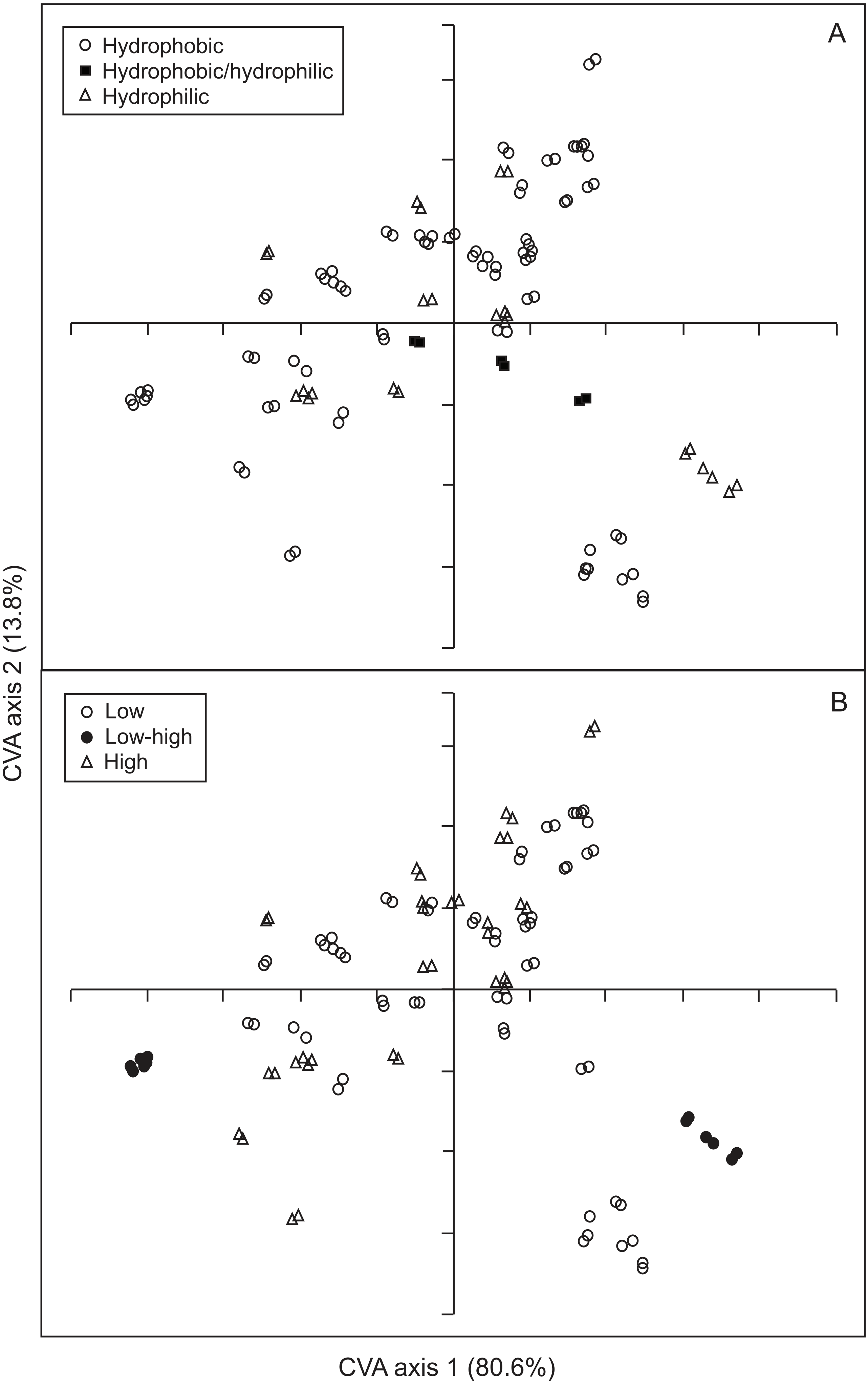
Hydrophobic
Hydrophobic/hydrophilic
Hydrophilic
A
Low
Low-high
High
B
CVA axis 2 (13.8%)
CVA axis 1 (80.6%)

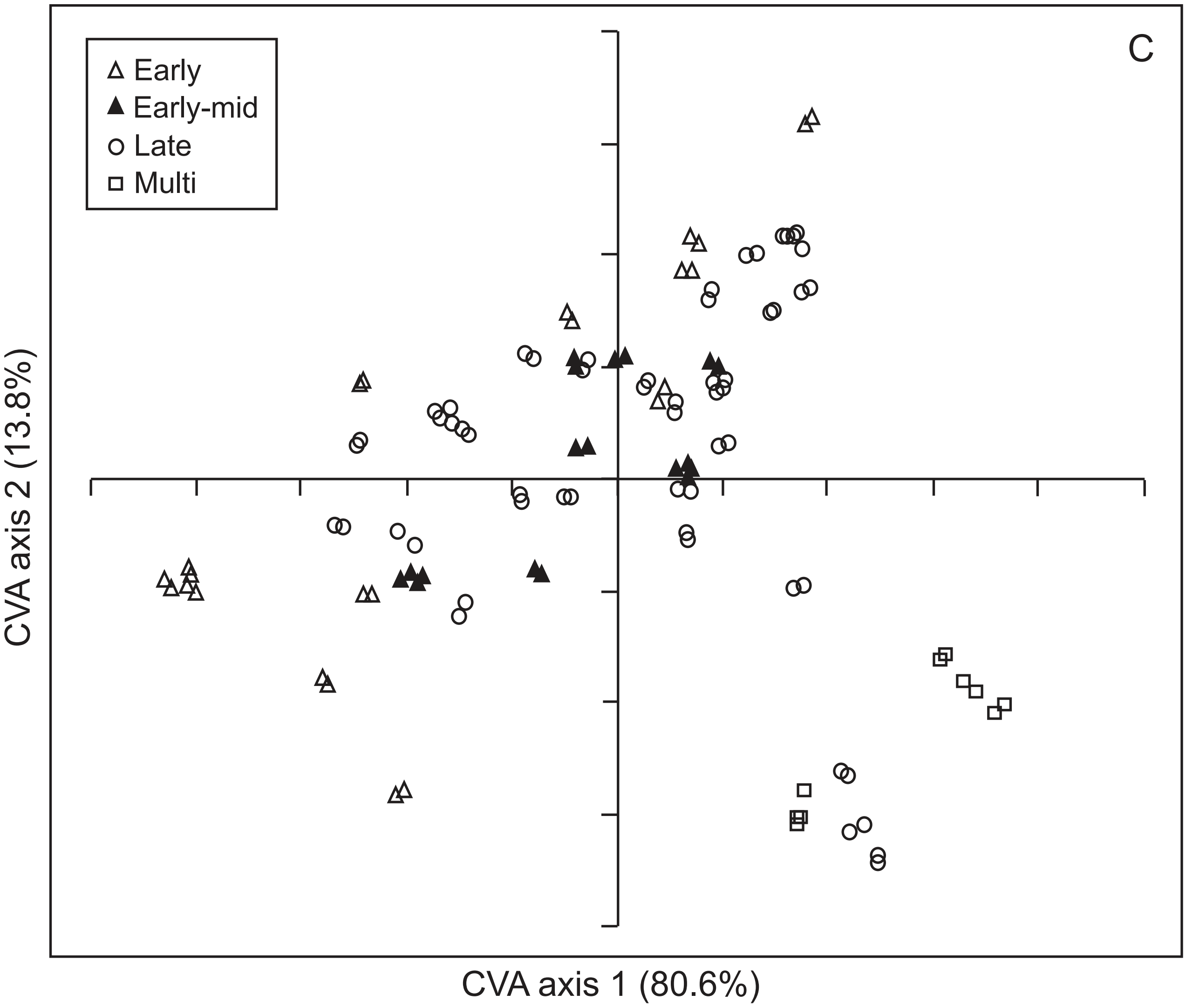


**Supplementary Fig. 6.** Separation in multivariate space was significantly explained by (A) hydrophobicity (MRPP: A = 0.112, P = 0.001), (B) N tolerance (MRPP: A = 0.08632, P = 0.001) and (C) succession (MRPP A = 0.05828, P = 0.001) when ECM fungal species were classified according to their putative ecological role and functional traits. Canonical variate analysis of FTIR spectra for ECM fungal isolates grown at three different C:N ratios (10:1, 20:1 and 40:1; same CVA as Fig. 3). See Table 1 for more information about classifications.

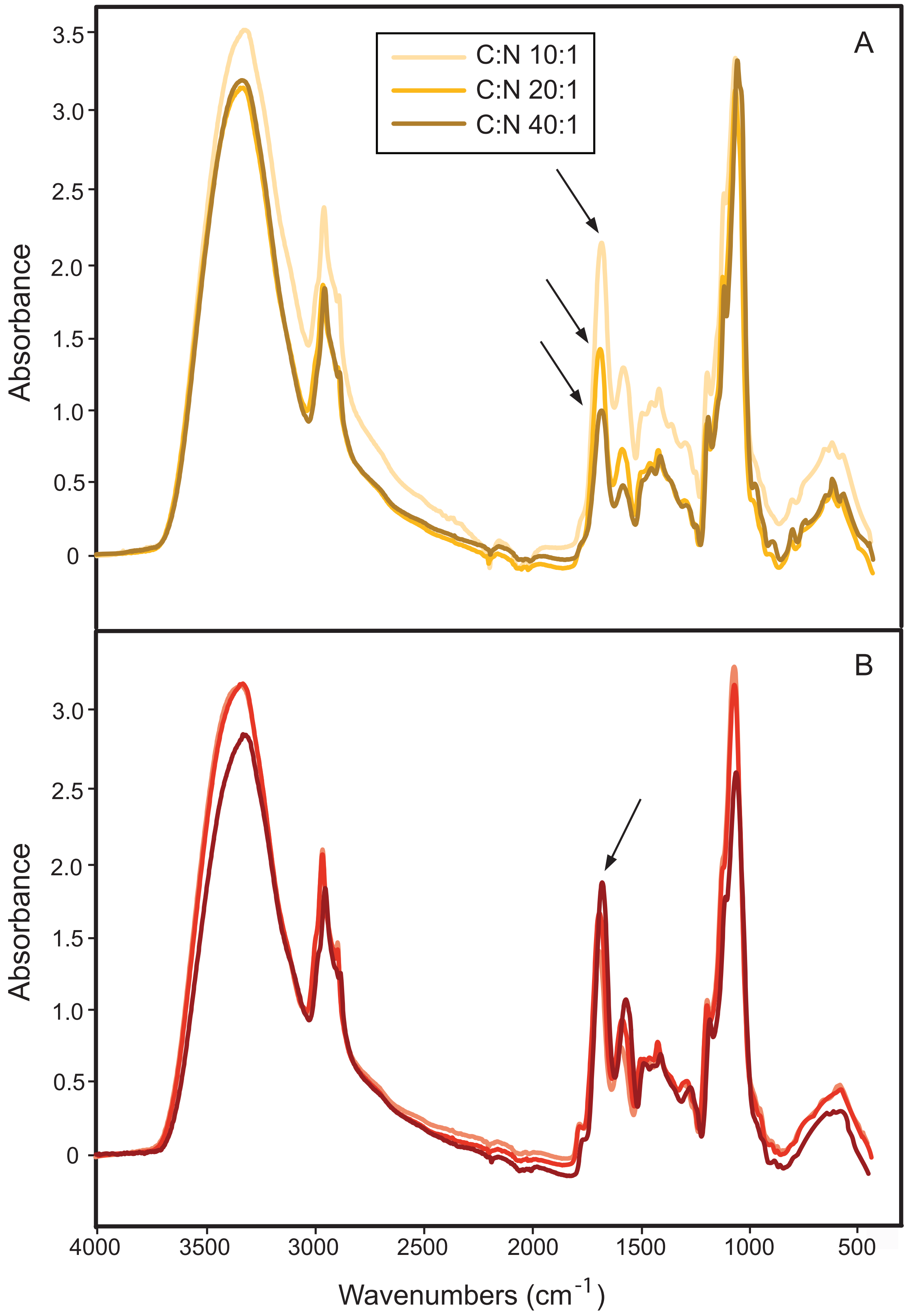

C:N 10:1
C:N 20:1
C:N 40:1
A
B
Absorbance
3.5
3.0
2.5
2.0
1.5
1.0
0.5
0
4000
3500
3000
2500
2000
1500
1000
500
Wavenumbers (cm$^{-1}$)

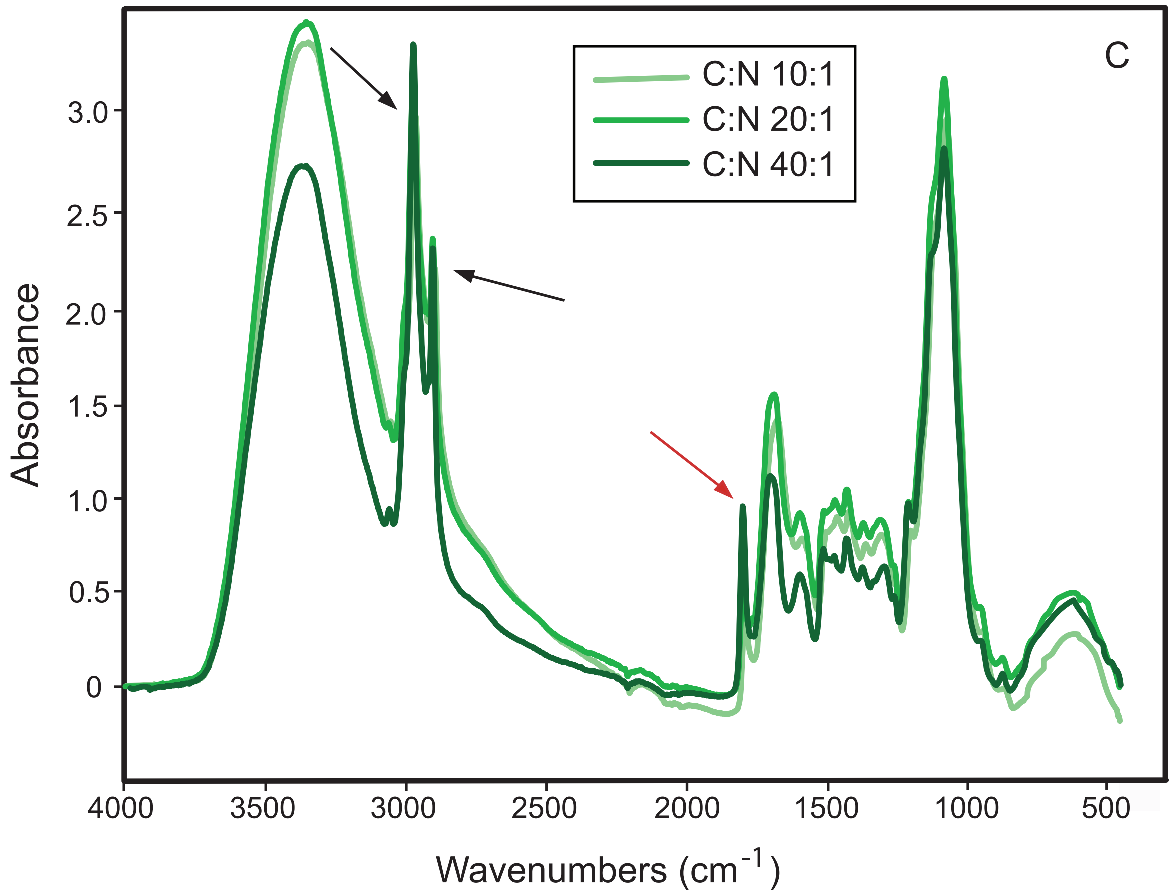


**Supplementary Fig. 7.** Changed carbon availability alters chemical composition of ECM fungi differently depending on species. FTIR spectra for (A) *A. muscaria*, (B) *L. bicolor* and (C) *P. bicolorata* mycelia grown at three different C:N ratios with an inorganic nitrogen source ($(NH_4)_2SO_4$). Arrows in (A) indicate reductions of amide peaks with increasing C:N ratio for *A. muscaria*. In (B) the arrow indicates the amide peak which was highest with C:N raio 40:1, and *L. bicolor* also represents a group of species together with *P. bicolorata* that showed increasing relative proportions of amide with increasing carbon availability. In (C) black arrows indicate large $CH_2$ peaks and the red arrow indicates the ester peak for *P. bicolorata*, showing subtle changes in waxy character in the C:N ratio 10:1. Absorbance (spectral signals) was normalised to give relative absorbance/abundance. Same data as shown off set in Fig. 5.

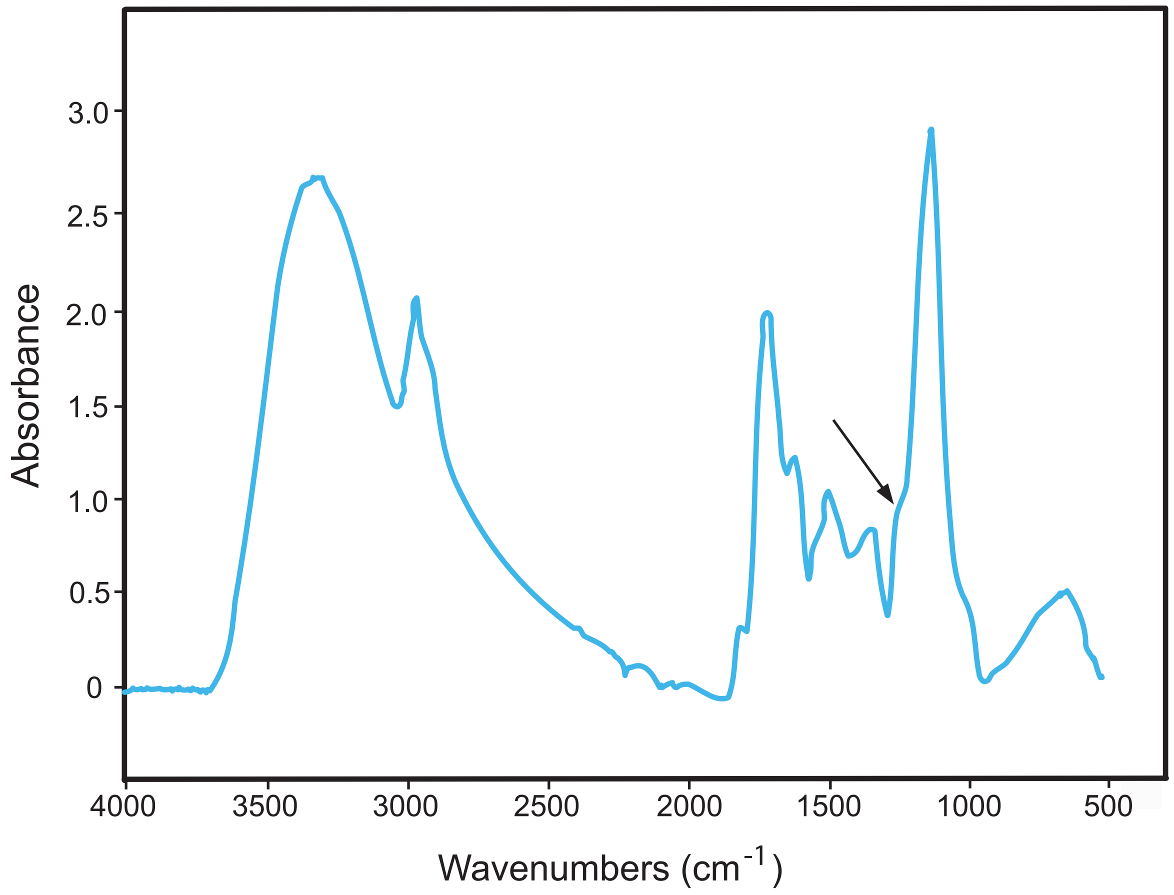


**Supplementary Fig. 8.** FTIR spectra for *P. fallax* mycelia grown at C:N ratio 40:1 with an inorganic nitrogen source ($(NH_4)_2SO_4$). *Piloderma fallax* was one of the species not responding much to carbon availability, instead chemical composition remained very similar. The arrow indicates a small oxalate peak. Absorbance (spectral signals) was normalised to give relative absorbance/abundance.
