# Supplementary material for: Carbon availability affects already large species-specific differences in chemical composition of ectomycorrhizal fungal mycelia in pure culture
Source: Mycorrhiza. 2023 Oct 12;33(5-6):303–19. doi: 10.1007/s00572-023-01128-2 (PMC10752919; doi:10.1007/s00572-023-01128-2)
Supplement: Supplementary file 2 — Supplementary file2 (PDF 1647 KB) [file 572_2023_1128_MOESM2_ESM.pdf]

Carbon availability affects already large species-specific differences in chemical composition of ectomycorrhizal fungal mycelia in pure culture

Mycorrhiza

Petra Fransson[1*], A.H. Jean Robertson[2] and Colin D. Campbell[2]

[1] Uppsala BioCenter, Department of Forest Mycology and Plant Pathology, Swedish University of agricultural Sciences , PO Box 7026, SE-75007 Uppsala, Sweden

[2] The James Hutton Institute, Craigiebuckler, Aberdeen AB15 8QH, Scotland

*** Correspondence:**

Petra Fransson e-mail: petra.fransson@slu.se

Supplementary Table 1. Reproducibility of ECM fungal spectra evaluated as average coefficient of variation for the two replicate IR spectra for each treatment. Replicates within treatments showed high consistency in chemical composition (see Fig. 3 and Fig. S1). Coefficient of variation (Coeff. Var.) and outliers corresponding to individual wavenumbers and the coefficient of variation (CV) values that were removed from the calculated averages per treatment are shown (n=2).

Species	C:N ratio	Coeff. var. (%)	Outliers (wavenumber [CV%])
Amanita muscaria	10	7,33	
Amanita muscaria	20	-12,44	
Amanita muscaria	40	-0,37	
Amphinema byssoides	10	2,30	
Amphinema byssoides	20	-11,39	1224.6 (241)
Amphinema byssoides	40	1,77	
Cenococcum geophilum	10	-2,12	
Cenococcum geophilum	20	-2,14	
Cenococcum geophilum	40	-1,23	
Cortinarius glaucopus	10	-10,62	2723.0 (1426)
Cortinarius glaucopus	20	4,44	1361.5 (1097), 1456.0 (2949)
Cortinarius glaucopus	40	-5,71	
Cortinarius scaurus	10	6,86	
Cortinarius scaurus	20	3,74	
Cortinarius scaurus	40	-0,11	
Hebeloma velutipes	10	-2,20	
Hebeloma velutipes	20	-3,86	
Hebeloma velutipes	40	-0,56	
Hebeloma sp. 1	10	-5,77	
Hebeloma sp. 1	20	-3,61	
Hebeloma sp. 1	40	-2,00	
Laccaria bicolor	10	4,19	
Laccaria bicolor	20	-3,61	
Laccaria bicolor	40	8,25	
Laccaria laccata	10	-5,75	
Laccaria laccata	20	1,65	
Laccaria laccata	40	2,17	
Paxillus involutus	10	-1,86	
Paxillus involutus	20	0,91	
Paxillus involutus	40	-5,29	
Piceirhiza biclorata	10	7,03	
Piceirhiza biclorata	20	-1,06	
Piceirhiza biclorata	40	-2,74	
Piloderma byssinum	10	-15,41	
Piloderma byssinum	20	-0,60	
Piloderma byssinum	40	-13,94	
Piloderma fallax	10	-5,88	
Piloderma fallax	40	0,06	
Rhizopogon roseolus	10	-6,14	1353.8 (238)
Rhizopogon roseolus	20	-0,05	

Rhizopogon roseolus	40	1,06	
Suillus bovinus BL	10	-1,97	3579.3 (3696)
Suillus bovinus BL	20	2,00	
Suillus bovinus BL	40	-2,46	
Suillus bovinus UP592	10	-4,25	
Suillus bovinus UP592	20	-1,81	1506.2 (1970)
Suillus bovinus UP592	40	-4,99	1353.8 (582)
Suillus bovinus UP63	10	-4,30	
Suillus bovinus UP63	20	-0,72	
Suillus bovinus UP63	40	-1,39	
Suillus variegatus UP597	10	-3,64	
Suillus variegatus UP597	20	-10,86	
Suillus variegatus UP597	40	-0,90	
Suillus variegatus UP60	10	-2,36	

Supplementary Table 2. Ectomycorrhizal mycelial biomass (average ± se) for 19 isolates grown at 25 degrees C for three weeks at three different C:N ratios (n=2). Different letters indicate significant differences between treatments (Two-way ANOVA, P=0.05).

**ECM fungal isolate**	**Biomass (mg)**		
	**C:N 10:1**	**C:N 20:1**	**C:N 40:1**
Amanita muscaria UP3	24.0±0.9 [ghijklmnopqr]	38.1±6.6 [cdefgh]	19.5±4.0 [klmnopqrstuv]
Amphinema byssoides A705	12.2±0.3 [opqrstuvw]	16.2±0.3 [lmnopqrstuvw]	18.8±0.6 [klmnopqrstuv]
Cenococcum geophilum Ve-95-12	12.2±0.3 [opqrstuvw]	17.5±2.1 [lmnopqrstuvw]	11.0±2.0 [qrstuvw]
Cortinarius glaucopus UP21	11.7±0.4 [opqrstuvw]	24.1±0.7 [ghijklmnopqr]	36.9±1.7 [cdefghi]
C. scaurus UP22	23.1±0.7 [hijklmnopqrs]	27.8±0.6 [fghijklmn]	35.0±0.1 [defghij]
Hebeloma velutipes UP184	3.3±0.1 [w]	11.3±0.9 [pqrstuvw]	17.2±0.3 [lmnopqrstuvw]
Hebeloma sp.1	10.6±0.6 [rstuvw]	18.8±0.4 [klmnopqrstuv]	26.0±0.5 [fghijklmnopq]
Laccaria bicolor CRBF581	5.4±0.1 [uvw]	21.6±0.8 [ijklmnopqrst]	38.7±1.5 [cdefg]
L. laccata Sk33	15.3±0.4 [mnopqrstuvw]	26.3±0.1 [fghijklmnopq]	29.3±3.7 [efghijklm]
Paxillus involutus G05	16.1±0.1 [lmnopqrstuvw]	30.8±2.3 [efghijkl]	50.4±1.0 [abc]
'Piceirhiza bicolorata' ARON2938.S	26.8±0.7 [fghijklmno]	49.4±0.1 [abcd]	43.9±9.1 [bcde]
Piloderma byssinum UP185	5.1±0.1 [vw]	13.1±1.8 [nopqrstuvw]	20.6±1.4 [jklmnopqrstu]
P. fallax UP113	8.4±1.8 [stuvw]	8.8±0.4 [rstuvw]	13.7±2.1 [nopqrstuvw]
Rhizopogon roseolus UP175	17.5±0.2 [lmnopqrstuvw]	39.5±0.4 [bcdef]	60.5±9.4 [a]
Suillus bovinus BL	3.2±0.4 [w]	13.7±2.8 [nopqrstuvw]	54.3±5.6 [ab]
S. bovinus UP63	7.1±0.3 [tuvw]	26.4±0.1 [fghijklmnop]	39.7±5.8 [bcdef]
S. bovinus UP592	5.1±0.1 [vw]	4.9±1.9 [vw]	36.3±3.0 [cdefghi]
S. variegatus UP60	12.4±1.4 [opqrstuvw]	29.9±0.1 [efghijklm]	31.3±1.2 [efghijkl]
S. variegatus UP597	20.0±1.3 [jklmnopqrstuv]	33.1±0.5 [efghijk]	38.9±0.4 [cdefg]
